# Novel role for mineralocorticoid receptors in control of a neuronal phenotype

**DOI:** 10.1038/s41380-019-0598-7

**Published:** 2019-11-19

**Authors:** Katharine E. McCann, Daniel J. Lustberg, Emma K. Shaughnessy, Kelly E. Carstens, Shannon Farris, Georgia M. Alexander, Daniel Radzicki, Meilan Zhao, Serena M. Dudek

**Affiliations:** 1 Synaptic and Developmental Plasticity Group, Neurobiology Laboratory, National Institute of Environmental Health Sciences, National Institutes of Health, 111 T.W. Alexander Drive, Research Triangle Park, NC 27709 USA; 2 Present Address: Molecular and Systems Pharmacology Graduate Program, Emory University, Atlanta, GA USA; 3 Present Address: Neuroscience Graduate Program, Georgia State University, Atlanta, GA USA; 4 Present Address: Center for Neurobiology Research, Fralin Biomedical Research Institute, Virginia Tech Carilion, Roanoke, VA USA

**Keywords:** Neuroscience, Cell biology, Physiology, Molecular biology

## Abstract

Mineralocorticoid receptors (MRs) in the brain play a role in learning and memory, neuronal differentiation, and regulation of the stress response. Within the hippocampus, the highest expression of MRs is in area CA2. CA2 pyramidal neurons have a distinct molecular makeup resulting in a plasticity-resistant phenotype, distinguishing them from neurons in CA1 and CA3. Thus, we asked whether MRs regulate CA2 neuron properties and CA2-related behaviors. Using three conditional knockout methods at different stages of development, we found a striking decrease in multiple molecular markers for CA2, an effect mimicked by chronic antagonism of MRs. Furthermore, embryonic deletion of MRs disrupted afferent inputs to CA2 and enabled synaptic potentiation of the normally LTP-resistant synaptic currents in CA2. We also found that CA2-targeted MR knockout was sufficient to disrupt social behavior and alter behavioral responses to novelty. Altogether, these results demonstrate an unappreciated role for MRs in controlling CA2 pyramidal cell identity and in facilitating CA2-dependent behaviors.

## Introduction

In the brain, glucocorticoid and mineralocorticoid receptors (GRs and MRs, respectively) mediate learning and memory, emotional states, and behavioral and physiological responses to stress. In most cells, including neurons, GRs and MRs act as transcription factors that control vast downstream networks of other transcriptional regulators, so activation of either receptor by the endogenous ligands cortisol or corticosterone (CORT) initiates complex programs of gene expression and repression [1–3]. Although both nuclear receptors recognize the same specific DNA promoter sequences called glucocorticoid response elements [4, 5], GRs and MRs control distinct transcriptional networks, which regulate diverse functions in neurons, including apoptosis, differentiation, and survival [5–13].

In contrast with GRs, which are expressed in virtually all cell types in the rodent brain, MRs are expressed primarily in neurons of limbic regions such as the hippocampus, lateral septum, and amygdala [10, 12, 14]. MRs have a tenfold higher affinity than GRs for CORT and are therefore thought to be occupied by ligand even under baseline, low-stress conditions [15, 16]. GRs, on the other hand, are activated when an animal is stressed or during circadian periods when circulating CORT levels are naturally elevated [5, 17, 18]. GR- and MR expression in hippocampal neurons is essential for normal regulation of the hypothalamic-pituitary-adrenal (HPA) axis, as GR- and MR-expressing neurons detect levels of circulating CORT and inhibit the HPA axis to terminate the stress response. Autoregulation of both MRs and GRs during this process, therefore, permits normal adaptations to stress [17–21].

In the hippocampal *Cornu Ammonis* (CA) fields, MR protein and mRNA *(Nr3c2)* are both strongly expressed by pyramidal cells (PCs) during embryonic development [22, 23] and remain enriched in hippocampal PCs throughout perinatal life and into adulthood [23–25]. Although multiple reports have demonstrated that MR expression in the hippocampus is highest in area CA2, beginning embryonically and lasting through adulthood [23, 25, 26], the biological significance of this early and concentrated MR expression in CA2 neurons remains unknown. CA2 pyramidal cells are further distinguished from neighboring CA1 and CA3 PCs in that they exhibit a unique pattern of gene expression that permits tight regulation of synaptic plasticity at CA3 → CA2 Schaffer collateral synapses [27] and confers sensitivity to the social neuropeptides oxytocin and vasopressin [28, 29], which may play a role in social recognition memory and aggression in mice [28–32]. Recent studies have demonstrated that CA2 PCs are important for social memory, aggression, spatial processing, and detection of novelty, behaviors that have also been linked to MRs [28, 32–36]. In this study, we set out to investigate whether deletion of MRs affects CA2 neuron physiology and function. Unexpectedly, we found that MR deletion resulted in a complete loss of CA2 molecular identity; all currently known molecular identifiers of CA2 drastically decreased in expression following deletion of MRs. Further, both widespread neuronal deletion and CA2-targeted deletion of MRs were sufficient to impair behaviors attributed to CA2 function. These findings demonstrate a defining role for MRs in the acquisition of CA2 pyramidal neuron fate, maintenance of CA2’s molecular profile, and expression of CA2-dependent behaviors.

## Results

### MR:GR ratio is highest in hippocampal area CA2

**Fig. 1:**
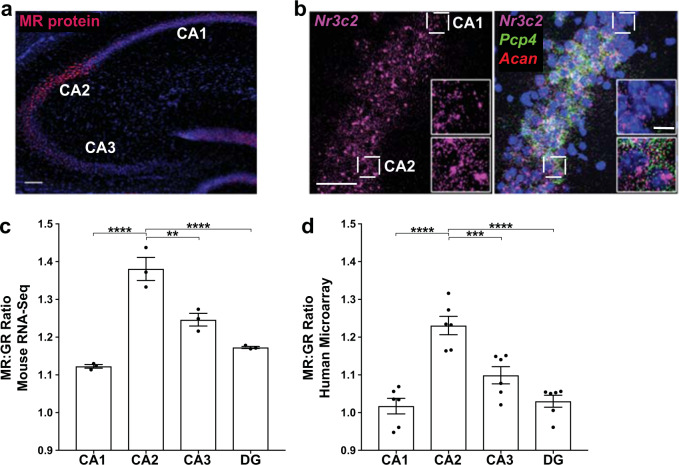
MR:GR ratio is highest in CA2. **a** Representative image of nuclear MR protein immunofluorescence in adult mouse hippocampus (scale bar = 100 μm). **b** Large transcriptional foci of MR message (*Nr3c2*, magenta) visualized using single-molecule fluorescent in situ hybridization (left; scale bar = 50 μm) co-expressed with the CA2 genes *Pcp4* (green) and *Acan* (red) (right). Insets show MR foci in CA2 (bottom insets) compared with MR foci in CA1 (top insets) (inset scale bar = 5 μm). DAPI nuclear stain is in blue for all images. Refer to Supplemental Fig. 3 for bregma coordinates. **c** RNA-Seq analysis of mouse hippocampal tissue indicated that the MR:GR mRNA ratio in adult CA2 was significantly higher than in each of the other hippocampal regions (one-way ANOVA, *F*(3,8) = 40.51, *p* < 0.0001; Bonferroni’s post hoc comparisons: CA2-CA1 *p* < 0.0001, CA2-CA3 *p* = 0.002, CA2-DG *p* < 0.0001; *n* = 3 mice). **d** Microarray data from the Allen Brain Institute showed that the MR:GR ratio in human hippocampus was also highest in CA2 compared with all other hippocampal regions (one-way ANOVA, *F*(3,20) = 21.39, *p* < 0.0001; Bonferroni’s post hoc comparisons: CA2-CA1 *p* < 0.0001, CA2-CA3 *p* = 0.0008, CA2-DG *p* < 0.0001; *n* = 6 human samples). **p < 0.01, ***p < 0.001, ****p < 0.0001

In the mouse hippocampus, the distribution of MRs and GRs is subregion specific. Staining for MRs can be observed by embryonic day (E)16.5 and is highly concentrated in CA2 by E18.5 [23], lasting into adulthood. We also observed this staining pattern in adult mice using an antibody recognizing the nuclear (transcriptionally active) state of the MR protein [37]. In adult mice, we found the highest density of MRs in area CA2 compared with all other hippocampal subregions (Fig. 1a). In addition, visualization of the gene encoding MRs, *Nr3c2*, using single-molecule fluorescent in situ hybridization, confirmed that *Nr3c2* was co-expressed with known CA2 pyramidal cell markers, aggrecan (*Acan*) and purkinje cell protein 4 (*Pcp4*) (Fig. 1b). RNA-Seq analysis of laser-captured, subregion-specific hippocampal tissue provided quantitative evidence that mouse CA2 has the highest MR:GR ratio compared with CA1, CA3, and the dentate gyrus (DG) (Fig. 1c). This concentration of MRs in CA2 is not unique to mice and is observed in human brain as well. In fact, when compared to the mouse hippocampus, microarray data from human samples [38] showed a similar pattern of MR:GR ratio in the hippocampal subregions (Fig. 1d), suggesting that the role of MRs in CA2 in mice may have significant translational relevance to humans.

### Loss of MRs inhibits the development of CA2’s molecular profile

**Fig. 2:**
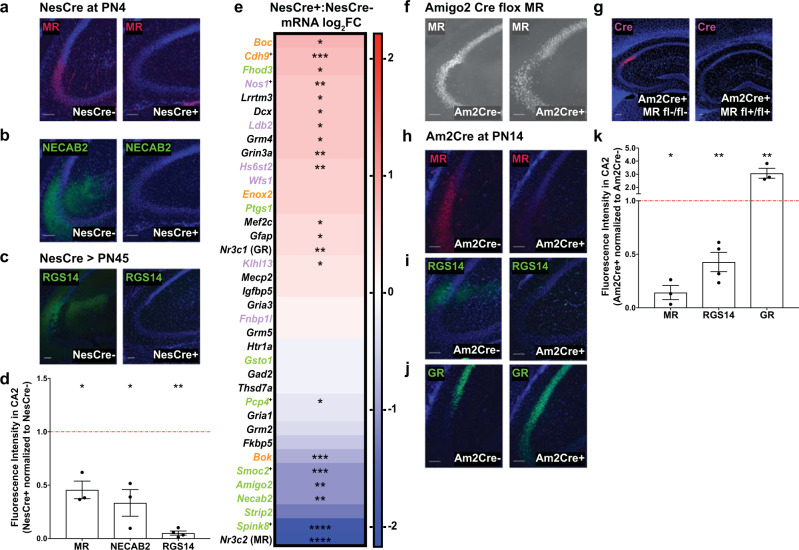
MR deletion disrupts CA2 molecular profile. **a** MR protein was detectable in CA2 at PN4 in control mice (left panel, NesCre^–^), but was not present in CA2 neurons of MR knockout mice (right panel, NesCre^+^ ). **b** NECAB2, an early marker of CA2 (left panel), was similarly not present in CA2 at PN4 of NesCre^+^ mice (right panel). **c** RGS14, a robust CA2 marker in adulthood, was not present in CA2 of NesCre^+^ mice older than PN45. DAPI nuclear stain is in blue for all images; scale bars = 100 μm. **d** Quantification of immunofluorescence data shown in **a–c**; data are normalized to NesCre^–^ mice (represented by red dashed line). Full statistics are shown in Supplemental Fig. 1. **e** NanoString mRNA analysis of hippocampal lysates at PN28 revealed 37 differentially expressed genes in NesCre^+^ compared with NesCre^–^ mice. All genes shown here differed with an uncorrected *p*-value of <0.05, and genes that met the adjusted *p*-value of < 0.05 are designated with *. This analysis confirmed several CA2 markers (green) were depleted after embryonic MR deletion, while several CA1 (purple) and CA3 (orange) markers were significantly higher after embryonic MR deletion. Differences are presented as log_2_ fold-change compared with NesCre^–^ mice. Genes that are abundant in more than one hippocampal region are designated with a plus symbol. **f** Representative images of MR protein expression in CA2 of Am2Cre^–^ (left) and Am2Cre^+^ (right) mice (PN28). **g** Representative image showing that cre was undetectable in CA2 of Am2Cre^+^ adult mice (right panel). The left panel shows cre staining in an Amigo2-cre line without floxed MR (MR fl^–^/fl^–^). **h–j** MR (**h**) and RGS14 (**i**) were decreased at PN14 after postnatal MR deletion (right panels), but GR staining (**j**) was increased in area CA2. **k** Quantification of immunofluorescence stains from PN14 shown in **h–j** are shown normalized to Am2Cre^–^ mice (represented by red dashed line). Full statistics are shown in Supplemental Fig. 1. DAPI nuclear stain is in blue for all color images. Scale bars = 100 μm. Refer to Supplemental Fig. 3 for bregma coordinates. *p < 0.05, **p < 0.01, ***p < 0.001, ****p < 0.0001

We first investigated how embryonic neuronal deletion of MRs would affect the development of CA2, specifically its gene and protein expression profiles. To do this, we crossed homozygous MR-floxed mice [39] with mice containing a *Nestin* promoter-driven cre recombinase (NesCre) to delete *Nr3c2* embryonically in the whole-brain (see Methods). MRs are already expressed in the hippocampus by embryonic day 16.5 [23], after the expected onset of cre expression in NesCre mice. Neuronal loss of MR protein was confirmed at postnatal day (PN)4 (Fig. 2a, d and Supplemental Fig. 1). Surprisingly, N-terminal EF-hand calcium binding protein 2 (NECAB2), which we have found to be one of the earliest markers of area CA2, was significantly reduced in NesCre^+^ mice (Fig. 2b, d and Supplemental Fig. 1) at PN4. This apparent loss of early CA2 molecular identity in NesCre^+^ mice continued throughout development. Regulator of G protein signaling 14 (RGS14) is a robust marker of CA2, starting by PN14 [27, 40], and staining for this protein was also severely diminished in NesCre^+^ mice, demonstrated here in mice older than PN45 (Fig. 2c, d and Supplemental Fig. 1). Furthermore, analysis of hippocampal mRNA at PN28 confirmed that several genes typically enriched in CA2 were significantly decreased (Fig. 2e, blue) in hippocampal tissue from NesCre^+^  mice compared with NesCre^–^ mice, and, interestingly, that several genes primarily expressed in CA1 and CA3 were increased (Fig. 2e, red and Supplemental Fig. 2). Staining for CA2 markers was also lost in adult mice with an embryonic knockout of MR using an *Emx1* promoter-driven cre recombinase (Supplemental Fig. 3).

Another defining characteristic of CA2 is glutamatergic inputs from the supramammillary nucleus of the hypothalamus (SuM), and these axon terminals can be labeled with antibodies raised against VGLUT2 [41]. Therefore, we next sought to determine whether embryonic deletion of MRs disrupted the targeting or maintenance of SuM axons, reasoning that if this projection requires MR-dependent expression of a growth factor or cell adhesion molecule, for example, we should detect a reduction in VGLUT2 staining. Indeed, we found that embryonic neuronal deletion of MRs resulted in significantly reduced VGLUT2 immunofluorescence in CA2, indicating a loss of SuM inputs to CA2 (Supplemental Fig. 4). In fact, in several of these knockout mice, we failed to detect any VGLUT2 expression in CA2. Interestingly, the fluorescence from the VGLUT2-positive axons imaged in the DG was also diminished when compared with NesCre^–^ mice (Supplemental Fig. 4). Notably, the putative (anatomical) CA2 region of mice with embryonic loss of MR still showed the characteristically disperse DAPI-stained nuclei and large cell bodies, indicating that at least some of the anatomical features associated with CA2 remained intact despite loss of molecular identifiers. These results indicate that MRs are necessary for acquisition of the CA2 molecular profile and suggest a novel role for MRs in the regulation of hippocampal development.

To further assess the role of MR expression in CA2 gene expression and to determine whether the reduction of CA2-enriched genes was due to a cell-autonomous loss of MRs, we crossed homozygous MR-floxed mice with mice expressing an *Amigo2* promoter-driven cre recombinase (Am2Cre) (see Methods). This line primarily targets cre to CA2 pyramidal neurons [42] and deletes MRs during postnatal development before adulthood (Fig. 2f and Supplemental Fig. 3). Interestingly, the cre recombinase itself was no longer detected in adult Am2Cre^+^ animals (Fig. 2g), suggesting that expression of cre driven by the *Amigo2* promoter, is also under transcriptional control of MRs, thus disappearing after cre-mediated deletion of MRs in CA2. This explanation is consistent with our mRNA analyses, showing that *Amigo2* expression was lower in NesCre^+^ mice compared with NesCre^–^ littermates (Fig. 2e and Supplemental Fig. 2). At PN4, neither MR nor NECAB2 immunoreactivities were significantly altered in sections from Am2Cre^+^ mice (Supplemental Figs. 1 and3), consistent with a postnatal onset of the conditional knockout. By PN14, MRs were undetectable in CA2 neurons of Am2Cre^+^ mice (Fig. 2h, k and Supplemental Fig. 1). In addition, Am2Cre^+^ mice had a significant reduction in the expression of all tested CA2 markers at PN14, including RGS14 (Fig. 2i, k and Supplemental Fig. 1), as well as NECAB2, PCP4, and a perineuronal net (PNN) marker, *Wisteria floribunda* lectin (WFA) (Supplemental Fig. 1). Furthermore, CA1 markers, including GRs (Fig. 2j, k) and wolframin ER transmembrane glycoprotein (WFS1) (Supplemental Fig. 1), exhibited increased immunoreactivity in putative CA2 neurons of Am2Cre^+^ mice at PN14. The striking changes in the CA2 gene and protein molecular profile, where CA2 markers were barely detected, if at all, and CA1 markers were increased in the region, were observed throughout development and maintained into adulthood (Supplemental Figs. 1, 2, and 3).

### CA2 proteins continue to be regulated by MRs in adulthood

**Fig. 3:**
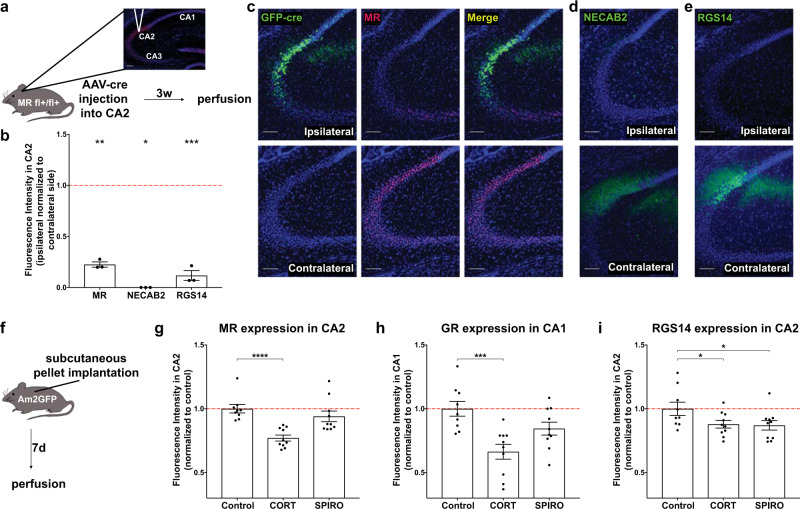
CA2 proteins continue to be regulated by MRs in adulthood. **a** Schematic of CA2-targeted unilateral rAAV5/hSyn-GFP-cre viral injection into adult MR fl^+^ /fl^+^ mice. **b** Quantification of MR, NECAB2, and RGS14 immunostaining from the ipsilateral (injected) side 3 weeks after unilateral injection (normalized to the contralateral, non-injected side, represented by red dashed line). **c** GFP-cre was contained to the ipsilateral CA2 and CA3a (top left panel) and was not present in the contralateral CA2 (bottom left panel). MR (middle panels) was not present in the CA2 neurons infected with GFP-cre virus. The right panel shows the merge of GFP-cre and MR fluorescence illustrating no overlap between the two. **d** NECAB2 and **e** RGS14 were significantly decreased in the ipsilateral CA2 (top panels) after a single GFP-cre injection when compared with the contralateral CA2 (bottom panels). Full statistics are shown in Supplemental Fig. 1. DAPI nuclear stain is in blue for all images. Scale bars = 100 μm. Refer to Supplemental Fig. 3 for bregma coordinates. **f** Schematic of subcutaneous pellet implantation of corticosterone (CORT, MR/GR agonist), spironolactone (SPIRO, MR antagonist), or vehicle control. **g** After 7 days of chronic CORT exposure, MR expression in CA2 decreased (one-way ANOVA: *F*(2,26) = 12.7, *p* = 0.0001; a priori LSD post hoc comparisons: CON-CORT *p* < 0.0001, CON-SPIRO *p* = 0.2224) and **h** GR expression in CA1 decreased (one-way ANOVA: *F*(2,26) = 9.018, *p* = 0.0011; a priori LSD post hoc comparisons: CON-CORT *p* = 0.0003, CON-SPIRO *p* = 0.0616). **i** While there was no main effect of drug (one-way ANOVA: *F*(2,26) = 3.249, *p* = 0.055), a priori post hoc comparisons showed that RGS14 expression in CA2 marginally decreased after 7 days of chronic CORT or chronic SPIRO exposure (a priori LSD post hoc comparisons: CON-CORT *p* = 0.0414, CON-SPIRO *p* = 0.0297). All quantifications are normalized to vehicle control, represented by red dashed line. *p < 0.05, **p < 0.01, ***p < 0.001, ****p < 0.0001

Next, to investigate if the observed molecular changes were solely a result of early developmental disruption of MRs or if MRs are also required for maintenance of the CA2 molecular profile, we introduced cre recombinase with an AAV virus injected unilaterally into CA2 of adult MR-floxed animals (Fig. 3a) and compared protein expression with the contralateral side within each animal. By 3 weeks post-injection, MR staining was eliminated from the ipsilateral (injected) CA2 (Fig. 3b, c), as was staining for other CA2 markers such as NECAB2 (Fig. 3b, d) and RGS14 (Fig. 3b, e), with no change in NeuN (Supplemental Fig. 1). These results indicate that at least some of the genes that make CA2 molecularly distinct are under transcriptional control of MRs not only during development, but also in adulthood.

In order to test for regulation of the CA2 molecular profile in a model of MR disruption in which MRs are merely modulated rather than knocked out, we implanted adult mice expressing green fluorescent protein (GFP) under the *Amigo2* promoter subcutaneously with slow-release pellets containing corticosterone (MR and GR agonist), spironolactone (MR-specific antagonist), or vehicle (Fig. 3f). After 7d, mice implanted with corticosterone had decreased MR staining in CA2 (Fig. 3g) and decreased GR staining in CA1 (Fig. 3h), consistent with previous work showing that autoregulated nuclear receptors are often downregulated when cells are in continuous presence of agonists [26, 43, 44]. Neither receptor was affected by the MR antagonist, spironolactone, at this time point (Fig. 3g, h). As would be expected if RGS14 was under the control of MR, RGS14 staining was decreased in mice implanted with corticosterone or with spironolactone (Fig. 3i). These data demonstrate that MR genetic deletion is not required to observe changes in CA2 protein expression and that pharmacological manipulation of MRs is sufficient to suppress CA2-specific gene expression.

### Embryonic MR deletion disrupts the CA2 synaptic plasticity phenotype

**Fig. 4:**
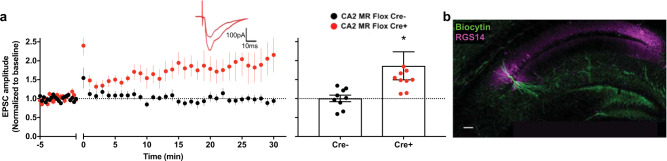
MRs are required during embryonic development for CA2’s plasticity-resistant phenotype. **a** Embryonic loss of MR (NesCre^+^) allowed for potentiation of CA2 excitatory postsynaptic currents (EPSCs) at PN14-18. EPSCs evoked with stimulation of the stratum radiatum are normally resistant to LTP pairing protocols (270 pulses at 3 Hz paired with postsynaptic depolarization, delivered at time = 0; NesCre^–^ mice (black)); however, this stimulation protocol resulted in significant potentiation of the EPSCs in CA2 neurons from NesCre^+^ mice (red). Inset traces from a representative NesCre^+^ experiment are the averages of responses evoked 5 min before, and the last 10 min after, pairing. Average EPSCs of the last 10 min were significantly higher in NesCre^+^ compared with NesCre^–^ mice (one-tailed *t*-test with Welch’s correction, *t*(9.972) = 2.265, *p* = 0.0235, *n* = 11 cells from six cre^+^ mice and *n* = 10 from six cre^–^ mice; note that two cells did not recover past 20 min and therefore are not represented in the dot plot). One cell fell outside of axis parameters (5.12) and is therefore not shown on the bar graph. This cell fell within our inclusion criteria and was not excluded from analysis. Our significant effect, however, is not driven by this one point. When this point was excluded from analysis, the *p*-value remained significant at *t*(16) = 4.206, *p* = 0.0003. **b** Example of a filled neuron (biocytin, green) overlapping with CA2 marker, RGS14 (magenta), from a NesCre^–^ mouse. DAPI nuclear stain is in blue. Scale bar = 100 μm. *p < 0.05

We next investigated whether embryonic MR deletion disrupted the unique properties of synaptic plasticity in CA2. Long-term potentiation (LTP) is normally not observed in stratum radiatum of CA2 pyramidal neurons [45], and previous work in our lab has shown that either knockout of RGS14 or degradation of PNNs could permit LTP in this region [27, 46]. As predicted based on the loss of both RGS14 and WFA stain in the MR knockout mouse (NesCre^+^ ), embryonic neuronal loss of MRs enabled LTP in CA2 from P14-P18 mice (Fig. 4a). Excitatory postsynaptic currents (EPSCs) recorded from NesCre^+^ CA2 neurons following an LTP “pairing protocol” increased over baseline (180 + /– 0.05% baseline). Synaptic responses recorded from CA2 neurons from NesCre^–^ mice showed the typical resistance to the LTP pairing protocol (99.0 + /– 0.02% baseline). Intrinsic properties did not change in a way that could explain this enhanced plasticity (Supplemental Fig. 5). These results show that neuronal deletion of MRs leads to plasticity regulation at CA2 synapses that is more typical of CA1 synapses.

### CA2-targeted deletion of MRs is sufficient to disrupt social discrimination

**Fig. 5:**
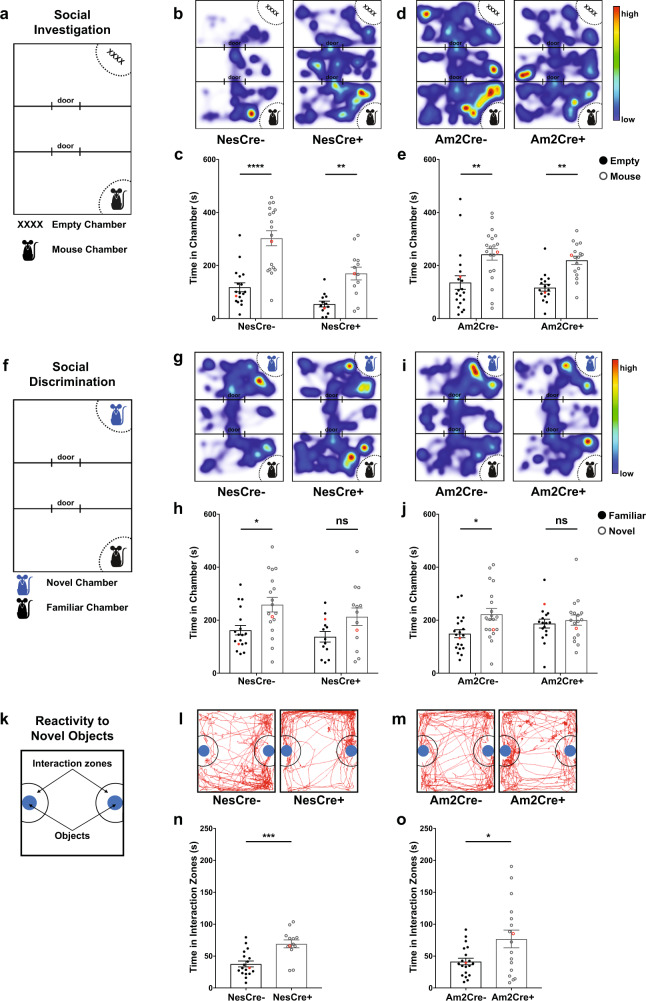
CA2-specific deletion of MRs is sufficient to drive behavioral changes in both social and non-social contexts. **a** After a 10 min habituation period to the testing arena, mice were tested for social investigation in a three-chamber task. During a 10 min period, mice were given the opportunity to explore the arena, one chamber containing a novel, same-sex conspecific (designated with black mouse icon) and the other chamber remaining empty (designated with xxxx). **b–e** A main effect of chamber was found for NesCre mice (**b**, **c**) (two-way rmANOVA, *F*(1,29) = 33.87, *p* < 0.0001) and for Am2Cre mice (**d**, **e**) (two-way rmANOVA, *F*(1,35) = 16.7, *p* = 0.0002). A main effect of genotype was also found in NesCre mice (*F*(1,29) = 4.511, *p* = 0.0423). Cre^+^ and cre^–^ mice from both strains showed a preference for the chamber containing the mouse (open circles in **c** and **e**) compared with the empty chamber (closed circles). Post hoc analysis for empty chamber vs. mouse chamber revealed these significant differences: NesCre^–^, *p* < 0.0001; Am2Cre^–^, *p* = 0.0043; NesCre^+^ , *p* = 0.0046, Am2Cre^+^, *p* = 0.0096. See Supplemental Table 1 for complete statistics. **f** After the social investigation task, a new novel mouse was placed in the once empty chamber to test mice for their ability to discriminate between a familiar (black mouse icon) and a novel (blue mouse icon) mouse. **g**–**j** During the social discrimination task, a main effect of chamber was found for NesCre mice, primarily driven by the cre-negative mice (**g**, **h**) NesCre: two-way rmANOVA, *F*(1,29) = 8.03, *p* = 0.0083). Post hoc analysis revealed that cre-negative mice of both strains showed a preference for the unfamiliar mouse (open circles in **h** and **j**) over the familiar mouse (closed circles) (NesCre^–^, *p* = 0.0204; Am2Cre^–^, *p* = 0.0313); however, neither **g**, **h** NesCre^+^ (*p* = 0.1131) nor **i**, **j** Am2Cre^+^ (*p* = 0.7126) mice showed this preference. See Supplemental Table 2 for complete statistics. Panels **b**, **d, g**, and **i** show representative heatmaps of the duration of time spent in each chamber, and the quantification of all mice are shown in **c**, **e, h**, and **j**, with the representative mice designated in red. **k** Mice were tested for their reactivity to novel objects in a familiar testing arena. After 2 days of habituation to the testing arena (10 min each day), on Day 3 mice were given 10 min to explore 2 novel objects in the same familiar context. **l**, **n** NesCre^+^ and **m**, **o** Am2Cre^+^ mice spent more time in the investigation zones actively investigating the novel objects than did their cre-negative littermates (NesCre: two-tailed *t*-test, *t*(29) = 4.165, *p* = 0.0003; Am2Cre: two-tailed *t*-test with Welch’s correction for unequal variances, *t*(20.39) = 2.401, *p* = 0.026). Representative traces of movement around the objects are shown in **l** and **m**, with the representative animals designated in red in **n** and **o**. ^ns^*p* > 0.05, *p < 0.05, **p < 0.01, ***p < 0.001, ****p < 0.0001

In previous work using the CaMKII promoter-driven cre recombinase to excise *Nr3c2* in forebrain neurons, mice were shown to have deficits in social recognition memory and hyper-reactivity to novel objects [33, 36]. We therefore asked if the behavior of our mice with an embryonic neuronal MR deletion was similar to that of mice with forebrain-only deletions, and whether our postnatal, CA2-targeted MR deletion was sufficient to cause these behavioral deficits. Mice were tested in a variety of behavioral assays measuring their locomotor activity, reactivity to novelty, anxiety-like behavior, and social behavior. Recent evidence has demonstrated that CA2 pyramidal cell activity is necessary for several forms of social behavior and social recognition memory in particular [28, 35]. When presented with a three-chamber testing scenario to measure social investigation (Fig. 5a), both cre-negative control and MR knockout mice (either NesCre or Am2Cre) spent more time in the chamber containing a novel same-sex stimulus mouse than in the empty chamber (Fig. 5b–e), indicating that all mice retained their normal drive for social investigation regardless of genotype (Supplemental Table 1). Furthermore, as expected when given the option to investigate a familiar or a novel, unfamiliar mouse (Fig. 5f), NesCre^–^ and Am2Cre^–^ mice spent more time investigating the novel mouse than the familiar mouse, suggesting they had a social memory of the familiar mouse, or at least had some preference for investigating novelty (Fig. 5g–j). Interestingly, neither strain of MR knockout mice displayed a significant increase in the time spent near the novel stimulus mouse, which is typically interpreted as the subject mouse having an impaired memory for the familiar stimulus mouse or a deficit in recognizing the novelty of the new mouse, thus resulting in decreased discrimination between the novel and familiar stimulus mice [47] (Fig. 5g–j and Supplemental Table 2). No differences were observed in the overall total time spent in the chambers with a stimulus mouse, indicating that the knockout mice were not avoiding social interaction (by spending more time in the center neutral chamber), but rather not displaying a preference for one mouse over the other (Supplemental Table 2).

### CA2-targeted deletion of MRs is sufficient to drive increased behavioral response to novel objects

Similar to what was reported in the CaMKII-cre, MR-floxed mice [36], the NesCre^+^ and the Am2Cre^+^ mice exhibited hyper-reactivity when exposed to novel objects (Fig. 5k). This was evident from an increased amount of time these mice spent in the interaction zones around the objects (Fig. 5l–o). NesCre^+^ also traveled more and at a faster speed during the testing period than did their NesCre^–^ littermates (Supplemental Table 3). Am2Cre^+^ mice traveled significantly less than their Am2Cre^–^ littermates, but did not have a significant change in overall speed (Supplemental Table 3). Similar to what we observed when the novel objects were present during behavioral testing, in a novel open field (Day 1) and in a familiar open field (Day 2 habituation), embryonic MR knockout mice (NesCre^+^) exhibited hyper-reactivity, as measured by an increase in total distance traveled and a higher speed of movement when compared with their NesCre^–^ littermates (Supplemental Table 3). In addition, post hoc analysis revealed a sex difference in the NesCre^+^ group, with males traveling more and faster than females in the novel open field. The hyper-reactivity of NesCre^+^ mice in the novel open field on Day 1 was also accompanied by a decrease in the time spent in the center of the arena, suggestive of a potential anxiety-like phenotype [48] (Supplemental Table 3). We observed no significant differences in the open field on Day 1 or Day 2 in distance traveled, speed, or in the time spent in the center between Am2Cre^–^ and Am2Cre^+^ mice (Supplemental Table 3).

To further assess for the possibility of an anxiety-like phenotype in the MR knockout mice, we studied the mice in an elevated plus maze. In this task, we observed no difference in the amount of time spent in or the number of entries into the open arms between NesCre^–^ and NesCre^+^ mice (Supplemental Table 4). Am2Cre^+^ mice, however, spent significantly more time in the open arms than did Am2Cre^–^ mice (Supplemental Table 4), indicating that postnatal deletion of MRs in CA2, as seen in these Am2Cre^+^ mice, may have an *anxiolytic*-like effect. We observed no differences in total distance traveled, speed, or the number of entries into the open arms between cre^–^ and cre^+^ for either strain of MR knockout mice (Supplemental Table 4); however, within the NesCre^+^ group, females traveled more and faster than did males. Based on these data, we conclude that MR deletion targeted at CA2 is sufficient to drive the hyper-reactivity to novel objects behavioral phenotype seen in whole-brain and forebrain MR knockouts. The effects of MR deletion on anxiety are unclear in that they rely on the specific test (open field vs. elevated plus maze), and differ depending on whether the deletion is embryonic, affecting all neurons (NesCre^+^), or postnatal, targeting CA2 pyramidal neurons (Am2Cre^+^). Altogether, however, these studies provide evidence that MR deletion targeted to CA2 pyramidal neurons, is sufficient to produce some of the same behavioral deficits as the larger MR deletions using Nestin or CaMKII to drive expression of cre recombinase.

## Discussion

Here, we report on the functional role(s) of MR expression in mouse hippocampal area CA2 as assessed by conditional deletion of the receptor. Our findings demonstrate that MR expression is required for the acquisition of CA2-specific pyramidal neuron gene and protein expression, as well as for the maintenance of these expression patterns throughout development and into adulthood. Although the neurons comprising the anatomical CA2 were grossly intact following MR deletion, as observed with NeuN and DAPI stains, these cells failed to express the molecular markers that normally distinguish CA2 neurons from neighboring CA1 and CA3. In fact, both protein and mRNA analyses indicated that genes normally highly expressed in CA1 and CA3 increased in expression in MR knockout animals, suggesting that MRs are not only critical for the development and maintenance of CA2’s unique molecular profile, but also in suppressing CA1- and CA3-specific gene expression. Surprisingly, embryonic deletion of MRs also disrupted inputs from the SuM into both CA2 and DG, further defining their role in hippocampal development. Some of the CA2 proteins disrupted by MR deletion are also highly expressed in DG (e.g., PCP4), and a loss of these proteins in both regions may be suggestive of their role in facilitating the development of hippocampal inputs. While not reaching significance, VGLUT2 staining to visualize SuM inputs into CA2 was also trending down in Am2Cre^+^ mice, suggesting that these inputs may also be disrupted with a targeted postnatal deletion of MRs (Supplemental Fig. 4). Future studies will be needed to determine the relative roles of these effects on CA2 and SuM connectivity and those due to the loss of plasticity regulating genes in MR-dependent behaviors.

The dramatic changes in CA2’s defining SuM input and in its molecular profile were accompanied by the physiologically abnormal enabling of Schaffer collateral LTP in CA2 pyramidal cells in mice with embryonic deletion of MRs. This phenomenon has also been observed in mice deficient for the plasticity-restricting protein RGS14 and in hippocampal slices treated with an enzyme that degrades CA2 PNNs [27, 46]. We did not observe any changes in intrinsic cell properties (Supplementary Fig. 5), so a remaining question is whether the behavioral phenotypes of these mice (see Fig. 5) are due to altered plasticity phenotype in CA2, or due to deficits in the specific cellular responses to steroid hormones driving the behavior.

As expected because many hormone receptors become downregulated under constant activation [26, 43, 44], MR and GR protein staining both decreased following 7 days of chronic CORT exposure. The hippocampus is part of the negative feedback loop of HPA axis activation, and increasing CORT levels decreases gene expression of stress hormone receptors [44]. Perhaps surprisingly, chronic treatment with the MR antagonist spironolactone did not affect MR staining intensity in CA2 but it was sufficient to cause a decrease in RGS14 staining intensity. MRs are known to autoregulate, evident by a decrease in MR mRNA in a brain region-specific manner, including in CA2, after treatment with the MR-specific agonist aldosterone [43] and an increase in MR protein in the hippocampus after adrenalectomy that is abolished by treatment with corticosterone [26]. These results, together with our antibody recognizing the activated, nuclear form of MRs [37], suggest that MR-mediated transcription in CA2 may be ongoing in response to baseline levels of hormone. The activation of MRs in CA2 could also be independent of adrenal glucocorticoid release, as enzymes required to synthesize corticosterone are present in hippocampal tissue [49–51]. Thus, it remains possible that localized synthesis of corticosteroids within the hippocampus may activate MRs in CA2 independent of the HPA axis and global stress response.

Some clues concerning the function of MRs in the hippocampus come from pharmacological studies using specific agonists and antagonists of MRs, as well as studies using genetic techniques to manipulate MR expression. Pharmacological manipulations of MR signaling or conditional deletion or overexpression of MRs in the forebrain have pronounced effects on hippocampus-dependent spatial tasks [52, 53], anxiety [54], fear memory [55, 56], and aggression [34, 57, 58]. In addition, MRs are implicated in the recognition of and reactivity to novel objects and novel conspecifics [33, 36]. Our mice, with either embryonic, whole-brain deletion (NesCre^+^) or postnatal, CA2-targeted deletion (Am2Cre^+^) of the gene encoding MRs, display many of these same deficits in behaviors as previously described for mice with postnatal forebrain deletion (using the CaMKII promoter). Specifically, we observed increased reactivity to novelty and a loss of social preference for a novel mouse. These findings emphasize a role for MRs in CA2 in regulating behavior, with deficits from loss of MR being similar to previous observations of cognitive and social deficits in mice with silenced, or lesioned CA2 neurons [32, 35, 42, 59]. As both area CA2 [35, 60] and MR deletion/regulation [33, 36, 52] have been implicated in social memory and novelty detection, the intriguing possibility that MRs mediate the transcription of genes that enable CA2 PCs to integrate social and contextual information to guide behavior should be considered.

The role of MRs and/or CA2 in anxiety are likely to be complex and are possibly sex-dependent. NesCre^+^ mice traveled more, at a faster speed, and spent significantly less time in the center of a novel open field than did their cre-negative littermates. Within this group, males traveled more and faster than did females. These data suggest an increase in anxiety-like behavior in NesCre^+^ mice, with a potentially stronger effect in males. The same anxiogenic effects were not observed in the Am2Cre^+^ mice, indicating that these effects in NesCre^+^ mice are likely due to loss of MRs outside of the hippocampus or, alternatively, due to the loss of SuM inputs into CA2 in the NesCre^+^ mice, which might rely on a secreted factor from CA2 for their development. In fact, Am2Cre^+^ mice displayed reduced anxiety-like behavior in the elevated plus maze, spending more time in the open arms than did their cre-negative littermates. This is consistent with the finding that MR blockade with MR-specific antagonists can produce anxiolytic-like behavioral responses [54, 61]; thus, our results indicate that MRs specifically expressed in CA2 may be a significant contributor to these effects on anxiety-like behavior. Future studies manipulating MRs in a more time-dependent manner (i.e., viral mediated knockout of MRs in adulthood) are needed to further clarify the apparent role of MRs in CA2 in regulating anxiety-like behavior.

The results of this study show that many of genes that make CA2 unique in the hippocampus are under the transcriptional control of MRs, and that by deleting MRs embryonically, postnatally, or in adulthood, CA2 pyramidal neurons are drastically altered. This transformation is evident by the changes in protein and mRNA expression, disruption of SuM inputs into CA2, the presence of LTP in CA2, and behavioral changes observed in both social and non-social contexts. The requirement of a single, autoregulatory transcription factor for both the embryonic acquisition and adult maintenance of neuronal identity has been demonstrated in other model organisms and may in fact be a fundamental organizational principle for the nervous system [62–64]. Such “terminal selector” transcription factors orchestrate cell fate determination and promote continuous expression of cell type-specific genes throughout the lifetime of the animal [65]. Thus, based on our finding that MR expression in hippocampal area CA2 is necessary at each stage of a mouse’s life in order for this region to develop and function normally, we propose the novel and exciting hypothesis that MRs are at least one of many possible terminal selector transcription factors for CA2 pyramidal cell identity. Further molecular and pharmacological studies investigating the network of transcriptional regulators downstream of MRs will be required in order to fully understand how MR-dependent transcription drives CA2’s molecular and plasticity-resistant profiles during development and maintains its specific battery of genes and role in behavior in adulthood. In conclusion, the results of this study raise fascinating questions about the effects of steroid receptor signaling on genetic programming of the hippocampus at all life stages, and demonstrate a critical role for MRs in the establishment of CA2 identity and the expression of CA2-dependent behaviors.

## Material and methods

### Animals and breeding

All animal protocols were approved by the National Institute of Environmental Health Sciences Animal Care and Use Committee and are in accordance with the National Institutes of Health guidelines for care and use of animals. Mice were housed on a 12 h:12 h light-dark cycle with same-sex littermates, with up to five animals per cage. Mice were provided water and food ad libitum. All animals were crossed on a c57BL/6J background (Jax 000664). Homozygous floxed mineralocorticoid receptor (MR fl^+^/fl^+^) mice, with *lox*P sites flanking exons 5–6 [39], were crossed to the *Nestin*-cre line (B6.Cg-Tg(Nes-cre)1Kln/J, Jax 003771), the *Amigo2*-cre line (B6.-Nr3c2 < tm2Gsc > Tg(Amigo2-cre)8Ehs) [42], or to the *Emx1-*cre line (B6.129S2-Emx1tm1(cre)Krj/J, Jax 005628). All offspring were genotyped using Transnetyx (Cordova, TN). All cre-positive mice used in each experiment were homozygous for the MR-floxed gene. Both males and females were used for all experiments and were not analyzed separately, unless otherwise reported. For each experiment, a power analysis was completed to determine the number of mice needed per group for statistical comparisons. Groups were determined based on genotypes (cre^–^ or cre^+^). Exact animal numbers for each immunofluorescence experiment are shown in Supplemental Figs. 1 and 4. Animal numbers for all other experiments are reported in the text and/or main figures.

### Immunofluorescence imaging and analysis

Mice were sacrificed at several developmental time points to assess molecular changes as a result of MR deletion over the course of the lifespan. An early postnatal (PN4) time point was chosen to determine the specific effect of embryonic deletion of MR (NesCre) at a time before the postnatal deletion (Am2Cre) occurred. Another subsest of animals was sacrificed at PN14 because this is a critical window during which many CA2 markers, including PNNs, begin to express [40, 46]. Later time points (PN28 and after PN45) were chosen to look at the long-term effects of MR KO through adolescence and into adulthood. For immunofluorescence processing, mice were deeply anesthetized with Fatal Plus (sodium pentobarbital, 50 mg/mL; > 100 mg/kg intraperitoneal (IP) injection). Animals older than PN14 were immediately perfused using 4% paraformaldehyde in phosphate buffer (pH 7.4). Brains were harvested and post-fixed in 4% paraformaldehyde for a minimum of 24 h at 4 °C. Animals younger than PN14 were killed by decapitation and their brains immediately harvested and post-fixed in 4% paraformaldehyde for a minimum of 48 h, rocking at 4 °C. Brains were sectioned at 40 μm on a cryostat at –20 °C or on a vibratrome and stored free floating in phosphate-buffered saline (PBS) with 0.02% sodium azide at 4 °C until subsequent processing. Brains that were cryosectioned were placed in a 30% sucrose solution in PBS (pH 7.4) for a minimum of 3 days at 4 °C before sectioning. Hippocampal sections from three or four animals (see Supplemental Figs. 1 and 4) per genotype from a minimum of two different litters were used for each condition. For immunofluorescence, some sections were antigen retrieved by boiling in citrate buffer for 1 min before blocking. All sections were blocked in 5% normal goat serum PBS with 0.1% TritonX and 0.02% sodium azide for 1 h, then incubated in primary antibodies overnight rocking at 4 °C (Supplemental Table 5). Sections were washed 3x 10 min in PBS with 0.01% TritonX, incubated in an Alexa Fluor secondary probe for 2 h (Supplemental Table 5), washed again 3 × 5 min, and coverslipped with Vectashield Hardset Mounting Medium with a DAPI nuclear counterstain (Vector, Burlingame, CA).

All images for quantification were taken on a Zeiss Epifluorescence microscope. Quantifications were made using original image files with ImageJ (1.49 v) and analyzed by an investigator blinded to genotype. Background fluorescence was subtracted using an area measured from outside of the hippocampal pyramidal layer. A region of interest (ROI) was highlighted in area CA2 using molecular and anatomical markers with the same size ROI used for every image. A minimum of two hippocampal measurements were averaged for each animal. Fluorescence intensities were normalized to the control group (cre-negative in NesCre and Am2Cre experiments; contralateral side to the cre injection in AAV injection experiments; vehicle control in pellet implantation experiments) and analyzed with a two-sided, unpaired *t*-test. If the standard deviations of the two groups were not equal, Welch’s correction was implemented (Prism 7 for Mac OS X, v.7.0c). All quantifications are shown as mean ± SEM, with dots representing each individual animal. All post-processing of images was performed in ImageJ after quantification and applied equally across compared genotypes in accordance with ethical policies on image integrity and standards.

### Viral injections

For infusion of rAAV5/hSyn-GFP-cre virus (University of North Carolina Vector Core, Chapel Hill, NC, lot#AV6446B, titer:6.2 × 10^12^) mice were anaesthetized with ketamine (100 mg/kg, IP) and xylazine (7 mg/kg, IP), then placed in a stereotaxic apparatus. Before surgery, animals were administered buprenorphine (0.1 mg/kg subcutaneous) for pain relief. An incision was made in the scalp, a hole drilled over the target region for infusion, and a 27-ga cannula connected to a Hamilton syringe by a length of tube was lowered into the CA2 region of the left hippocampus (in mm: –2.3 AP, –2.4 ML, -1.9 DV from bregma). For each infusion, mice were infused unilaterally with 0.5 µl of rAA5V-hSyn-GFP-cre over a 5-min period. The cannula was left in place for an additional 10 min before removing. The scalp was sutured and the animals were returned to their home cage for recovery. Animals were group housed for 3 weeks then sacrificed for immunofluorescence following the protocol outlined above.

### NanoString sequencing

Mice at PN28 (±1 day) (*n* = 3 males from different litters and *n* = 3 females from different litters for each genotype and strain, *N* = 12) were deeply anesthetized with Fatal Plus as described above and brains were quickly harvested on ice in an RNase-free environment. A sagittal cut was made to separate the two hemispheres. Each hippocampus was dissected free and the ventral one-third (approximate) was removed. Hippocampal tissue was immediately frozen on dry ice and stored at –80 °C for subsequent processing. RNA was extracted using the Qiagen RNeasy Mini Kit (Germantown, MD), and the concentration of each sample was measured using a nanodrop spectrophotometer. mRNA levels were determined using the NanoString (www.nanostring.com) platform utilizing a custom Code Set that measured 109 endogenous RNAs and 11 housekeeping genes (Supplemental Table 6). One-hundred nanograms of each total RNA sample was prepared as per the manufacturer’s instructions. RNA expression was quantified on the nCounter Digital Analyzer and raw and normalized counts were generated with nSolver (v3.0) software. Data were normalized utilizing the manufacturer’s positive and negative experimental control probes, as well as three housekeeping genes with low %CV and that were representative of the range intensity of the endogenous data (*Ppia*, *Sdha*, and *Tbp*). All but one sample passed nSolver’s initial QA/QC controls (NesCre-negative female), and this sample was omitted from analysis. All replicates were well correlated (*R* > 0.95). Normalized data (log_2_ of counts) were imported into Partek (St. Louis, MO) and quantile normalized for further QA/QC and statistical analyses. To identify significant differences in RNA expression, a *t*-test on treatment groups was performed with post-hoc Benhamini-Hochberg false-discovery rate for each group comparison. Six low-expressing genes from the custom code set did not quantify above noise and therefore were not included in further analysis (indicated in Supplemental Table 6).

### Electrophysiology

Mice, either NesCre^–^ or NesCre^+^, were used; in some cases, but not all, investigators were blind to the genotype of the animals. Animals were deeply anesthetized with Fatal Plus as described above, decapitated, and their brains removed and submerged into oxygenated ice-cold sucrose-substituted artificial cerebrospinal fluid (ACSF) of pH 7.4 (in mM: 240 sucrose, 2.0 KCl, 1 MgCl_2_, 2 MgSO_4_, 1 CaCl_2_, 1.25 NaH_2_PO_4_, 26 NaHCO_3_ and 10 glucose). Brain slices were cut coronally at 300 μm using a vibrating microtome (Leica VT 1000 S) and allowed to recover at room temperature in a submersion holding chamber with ACSF (in mM): 124 NaCl, 2.5 KCl, 2 MgCl_2_, 2 CaCl_2_, 1.25 NaH_2_PO_4_, 26 NaHCO_3_, and 17 d-glucose bubbled with 95% O_2_ with 5% CO_2_. Whole-cell recordings were made from pyramidal neurons in area CA2, which were identified visually using differential interference contrast (DIC) optics (CA2 neurons were verified in earlier, separate experiments using a CA2-specific fluorescent reporter mouse line). Glass borosilicate pipettes were filled with a potassium gluconate internal solution (in mM: 120 K-gluconate, 10 KCl, 3 MgCl_2_, 0.5 EGTA, 40 HEPES, 2 Na_2_-ATP, 0.3 Na-GTP, pH 7.2), with a tip resistance between 3–4.5 MOhms. Data were collected using Clampex 10.4 and analyzed using Clampfit software (Axon Instruments). Series and input resistances were monitored by measuring the response to a 10 mV step at each sweep and cells were included for analysis if series and input resistance changed by < 30%. Recordings were not compensated for series resistance. To assess excitatory transmission in P14-18 slices, whole-cell recordings were performed in voltage-clamp mode, and postsynaptic currents (PSCs) were evoked with a bipolar cluster electrode (FHC, #CE2C75) placed in stratum radiatum. For long-term potentiation (LTP) experiments, baseline excitatory PSCs (EPSCs) were collected every 15 s for at least 5 min after which a pairing protocol was used, consisting of 1.5 min of 3 Hz presynaptic stimulation (270 pulses) paired with postsynaptic depolarization to 0 mV in voltage-clamp mode. Data were averaged and normalized to baseline. Biocytin was used to fill CA2 neurons to show representative neurons targeted in all LTP experiments (Fig. 4b). Note that it was not possible to positively identify CA2 neurons in slices from NesCre^+^ mice, as CA2 markers are undetectable and CA1 markers invade the location of the CA2 region.

### Single-molecule fluorescent in situ hybridization and image acquisition

Animals were deeply anaesthetized with Fatal Plus as described above, followed by rapid decapitation. Brains were harvested and frozen in Tissue Plus® O.C.T Compound (Fisher Scientific, Hampton, NH) over dry ice. The brains were cryosectioned at 20 μm and mounted on SuperFrost® Plus slides (Fisher Scientific). The slides were then processed for single-molecule FISH according to the RNAscope Fluorescent Multiplex kit instructions (Advanced Cell Diagnostics, Hayward, CA). The following probes were used: *Nr3c2* (Cat#456321), *Pcp4* (Cat#402311), and *Acan* (Cat#439101). The slides were imaged on a Zeiss LSM 880 inverted confocal microscope with a 40x oil immersion lens. Acquisition parameters were set using 3plexed negative controls (complementary DNA probes against bacterial RNAs not present in mouse tissue) in each of the three channels (Alexa 488, Atto 550, Atto 647) so that any signal above the level of background was acquired. Area CA2 borders were defined using *Pcp4* as molecular marker.

### Mouse RNA-sequencing and human microarray data

RNA-Seq data were obtained from previously published work [66, 67] (Gene Expression Omnibus SuperSeries GSE116343). Briefly, laser capture microdissected samples (*n* = 3 per region) targeting the cell body region of CA1, CA2, CA3, and dentate gyrus were obtained and sequenced using an Illumina NextSeq 500 instrument, acquiring 100 bp paired-ends reads to a depth of 50 million reads per sample ( + /– 10 mil). Raw reads were pseudoaligned to GENCODE (vM12) gene models and the mm10 mouse genome and quantitated per gene using Salmon (v0.9.1, indexed with kmer size 31) [66–68]. The ratio of MR:GR was calculated from the normalized counts for each sample and averaged for each hippocampal subregion. A one-way analysis of variance (ANOVA) was conducted to compare the ratios, and a Bonferroni post-hoc analysis was used to determine significant differences between CA2 and each of the other regions (Prism 7 for Mac OS X, v.7.0c). Human microarray data were collected from the Allen Brain Institute [38]. Log_2_ expression from six donors was collected for MR and GR expression from each of the hippocampal subregions, CA1, CA2, CA3, and DG. The expression values for each region were transformed into an MR:GR ratio for each donor. A one-way ANOVA was conducted to compare the ratios, and Bonferroni post-hoc analysis was used to determine significant differences between CA2 and each of the other regions.

### Pellet implantation

Mice (*n* = 5 males and 5 females per group) were briefly anesthetized with 2.5% isoflurane and subcutaneously implanted with a 21-day release pellet (Innovative Research of America, Sarasota, FL, USA) containing either 15 mg corticosterone (Cat#G-111), 25 mg spironolactone (specific MR antagonist; Cat#M-161), or vehicle (Cat#C-111). After 7 days, animals were perfused and brains were processed for immunofluorescence, as described above. One control male was removed from the study because he removed his wound clips and pellet shortly after surgery. No sex differences were detected and therefore males and females were collapsed for analyses. Comparisons were made using a one-way ANOVA, with a priori LSD post hoc comparisons between the control group and each of the two drug groups.

### Behavioral testing

Animals (*n* = 32 NesCre, *n* = 40 Am2Cre, across five cohorts) were brought to the behavior room and allowed to habituate for a minimum of 20 min before each behavioral task. All behavioral experiments were performed during the light cycle. For each task, all subjects were run within the same 3 h circadian window. All mice were tested in each behavioral task with a minimum of 24 h between each task to minimize interference from previous HPA axis activation on subsequent tasks. Social assays were completed a minimum of 72 h after the last non-social task. The experimenter was blinded to genotype during all behavioral testing. Four animals were excluded from analysis for the following reasons: one Am2Cre^+^ male was inadvertently left out of the elevated plus maze, and therefore not included in any analyses; one Am2Cre^–^ male fell off the elevated plus maze and was removed from the study; one Am2Cre^+^ female jumped out of the arena during novel object testing and was removed from the study; one NesCre^–^ female was removed from the study as an outlier (*z*-score = 3.44 for both distance traveled and speed during sociability task). Final animal number per group were as follows: NesCre^–^ *n* = 18 (7 female, 11 male), NesCre^+^ *n* = 13 (7 female, 6 male), Am2Cre^–^ *n* = 20 (7 female, 13 male), Am2Cre^+^ *n* = 17 (6 female, 11 male). Post hoc comparisons between sexes were made within each group, and, unless otherwise reported, no sex differences were observed during any behavioral task.

The Elevated Plus Maze assay was performed first (Day 1). Each animal was placed on a clean elevated plus maze in the center and left to investigate for 5 min. The animal was removed after 5 min and returned to its home cage. Movement about the maze, including distance traveled, speed, and entries into and duration spent in the open arms were recorded using Noldus Ethovision software. Comparisons between cre-negative and cre-positive mice were completed using a two-tailed *t*-test. The maze was thoroughly cleaned with a hydrogen peroxide-based disinfectant between each animal.

The next day (Day 2, open field), animals were placed in a clean, empty novel arena and allowed to investigate for 10 min, after which they were returned to their home cage. The animals’ reactivity to the novel environment, including distance traveled, speed, and duration spent in the center of the arena, was recorded using Noldus Ethovision and compared between cre-negative and cre-positive mice using a two-tailed *t*-test. Each arena was thoroughly cleaned with an ammonia solution between each animal. The following day (Day 3, habituation), each animal was again placed in the same clean, empty arena and allowed to investigate for 10 min in order to habituate to the environment. On the fourth day, two identical novel objects were placed on the edges of the arena (Fig. 5k). The animals were returned to the same arena and allowed to investigate the arena and the objects for 10 min after which they were returned to their home cage. The animals’ movement about the arena and duration of time spent investigating the objects (“interaction zone”) were recorded using Noldus Ethovision and compared using a two-tailed *t*-test.

The following week, animals were tested for social investigation and social discrimination. Animals were placed in an arena with three chambers (with a door between the chambers) and a small perforated barrier in one corner of each of the top and bottom chambers. They were allowed to investigate the entire arena for 10 min. After 10 min, the mice were gently moved into the center chamber and the doors were closed. A novel sex- and age-matched conspecific was added behind one of the barriers (Fig. 5a). The doors were opened, and the subject animal was allowed to explore the arena. After 10 min, the animal was again gently moved to the center chamber and doors were closed. A second novel conspecific (sex- and age-matched from a different litter than the first) was added behind the remaining barrier (Fig. 5f). The doors were opened, and the subject animal was allowed to explore the arena for 10 min. The location of the stimulus animals in the top or bottom chamber was counterbalanced across subjects. At the end of the 30-min session, each animal was returned to its home cage. The arena was thoroughly cleaned with 70% ethanol before the next animal was tested. Each trial was recorded and tracked using Noldus Ethovision software. Time spent in each chamber of the arena was recorded and compared between cre-negative and cre-positive mice using a two-way, repeated-measures analysis of variance (rmANOVA).

## Supplementary information

Supplemental Information
