# Supplementary material for: Novel role for mineralocorticoid receptors in control of a neuronal phenotype
Source: Mol Psychiatry. 2019 Nov 19;26(1):350–64. doi: 10.1038/s41380-019-0598-7 (PMC7234915; doi:10.1038/s41380-019-0598-7)
Supplement: Supplementary file 1 — Supplemental Information [file 41380_2019_598_MOESM1_ESM.pdf]

Novel role for mineralocorticoid receptors in control of a neuronal phenotype

Supplemental Information

K. E. McCann, D. J. Lustberg, E. K. Shaughnessy, K. E. Carstens, S. Farris, G. M. Alexander, D. Radzicki, M. Zhao, and S. M. Dudek

Supplemental Figure 1

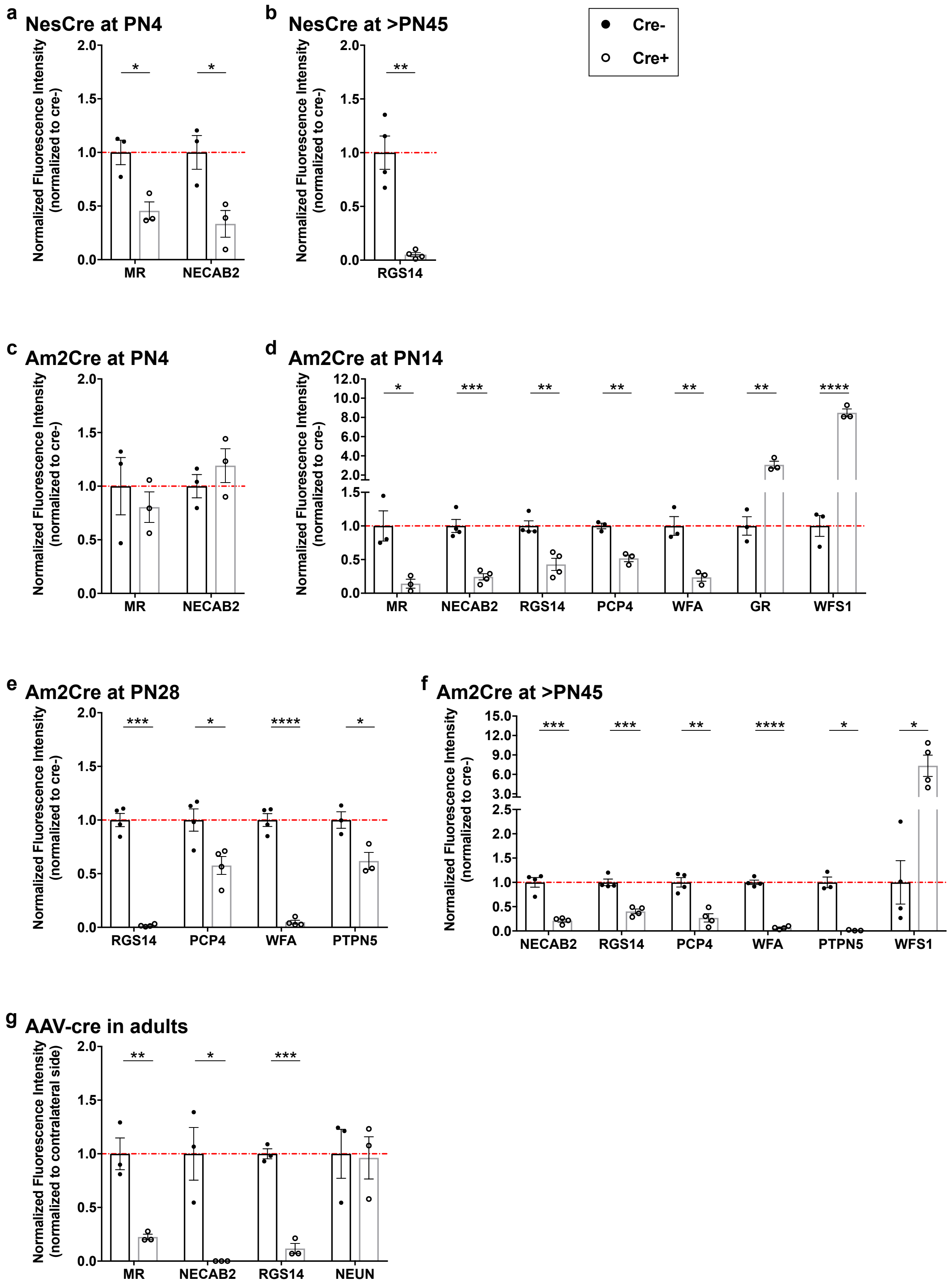

Supplemental Figure 1 Quantification and full statistics of each immunofluorescence stain*. Refers to Figures 2, 3, and Supplemental Figure 3. Quantifications were normalized to the average of cre- mice or to the contralateral (non-injected) CA2 (represented by red dashed line). Shown are data from NesCre at* ***(a)*** *PN4 and* ***(b)*** *>PN45 (refers to Figure 2); Am2Cre at* ***(c)*** *PN4,* ***(d)*** *PN14,* ***(e)*** *PN28, and* ***(f)*** *>PN45 (refers to Figure 2, Supplemental Figure 3); and* ***(g)*** *cre recombinase delivered by AAV in adults (refers to Figure 3).*

Cre-negative mice are represented by closed circles; cre-positive mice are represented by open circles. All comparisons were made using an unpaired, two-tailed t-test. Full statistics for each stain:

NesCre at PN4: MR t(4)=3.865, p=0.0181; NECAB2 t(4)=3.321, p=0.0293

NesCre at >PN45: RGS14 t(6)=6.085, p=0.0009, [+]p=0.0082

Am2Cre at PN4: MR t(4)=0.6438, p=0.5548; NECAB2 t(4)=1.003, p=0.3725

Am2Cre at PN14: MR t(4)=3.673, p=0.0213; NECAB2 t(6)=7.091, p=0.0004; RGS14 t(6)=4.868, p=0.0028; PCP4 t(4)=7.48, p=0.0017; WFA t(4)=5.047, p=0.0072; GR t(4)=5.166, p=0.0067; WFS1 t(4)=17.62, p<0.0001

Am2Cre at PN28: RGS14 t(6)=16, p<0.0001, [+]p=0.0005; PCP4 t(6)=3.197, p=0.0187; WFA t(6)=15.26, p<0.0001; PTPN5 t(4)=3.43, p=0.0265

Am2Cre at >PN45: NECAB2 t(6)=7.556, p=0.0003; RGS14 t(6)=7.222, p=0.0004; PCP4 t(6)=5.639, p=0.0013; WFA t(6)=19.85, p<0.0001; PTPN5 t(4)=9.041, p=0.0008, [+]p=0.0118; WFS1 t(6)=3.703, p=0.0101

AAV-cre in adults: MR t(4)=5.148, p=0.0068; NECAB2 t(4)=4.074, p=0.0152, [+]p=0.0553; RGS14 t(4)=13.16, p=0.0002; NEUN t(4)=0.1247, p=0.9068

[+]Denotes adjusted p-value after Welch's correction applied for unequal variance between groups.

**p<0.05, **p<0.01, ***p<0.001 ****p<0.0001*

Supplemental Figure 2

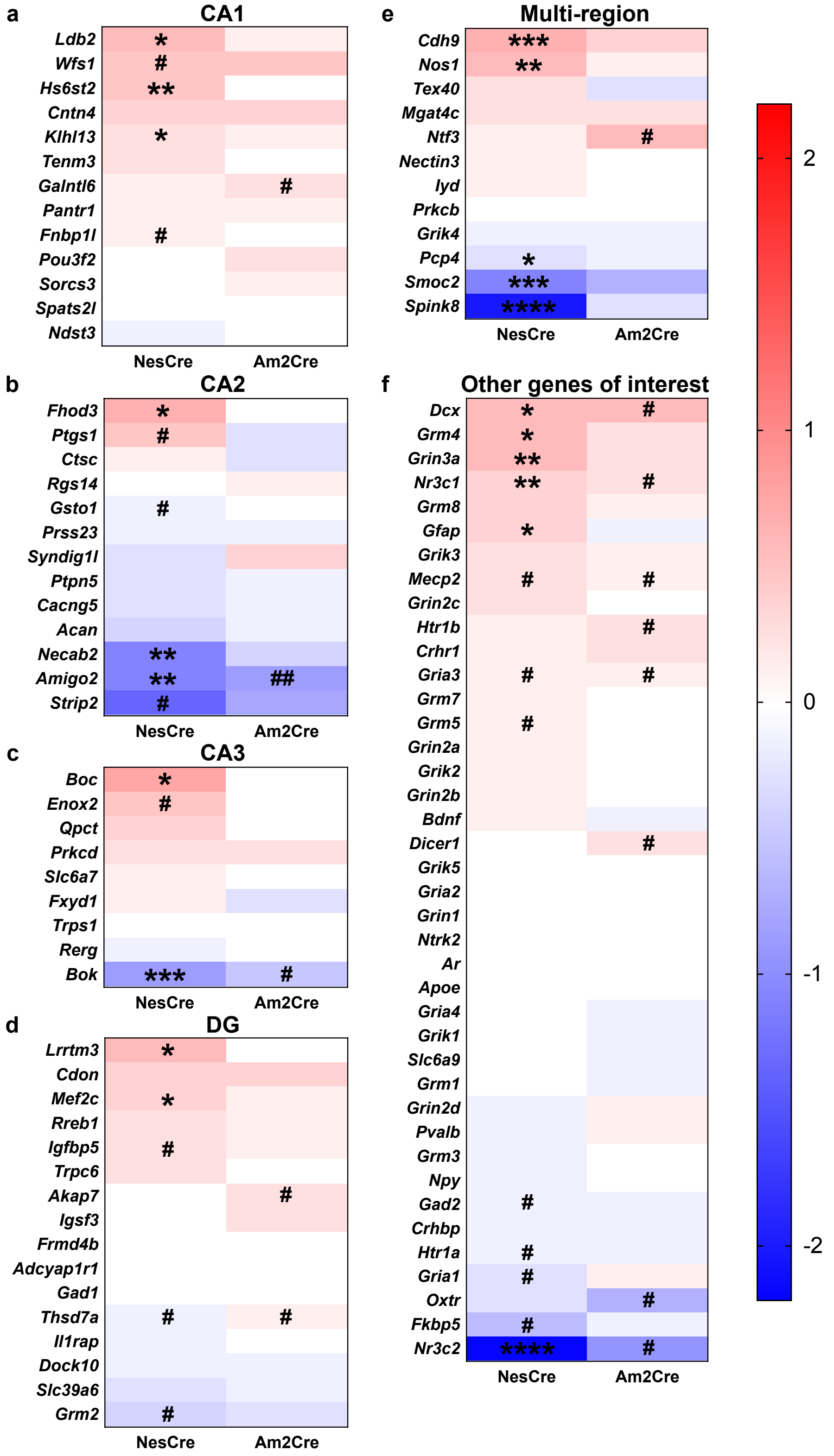

Supplemental Figure 2 Complete set of genes tested using NanoString analysis of whole hippocampal lysates. *Refers to Figure 2 and Supplemental Table 6.* ***(a)*** *Of the CA1 genes tested, several had higher expression (red tones) in NesCre+ tissue, while most of the* ***(b)*** *CA2 genes had lower expression (blue tones) or trended negative when compared with NesCre- tissue. Similar trends were observed in tissue from Am2Cre+ animals.* ***(c-e)*** *Genes enriched in* ***(c)*** *CA3 and* ***(d)*** *dentate gyrus (DG) were mixed in their expression changes, as were genes that are expressed in* ***(e)*** *multiple hippocampal regions.* ***(f)*** *We also assessed several additional genes of interest, including those encoding*

*glutamate receptors and gene regulated by stress. No genes reached significance in Am2Cre+ compared with Am2Cre- mice using an adjusted p-value to correct for multiple comparisons, likely due to the small size of the CA2 relative to other hippocampal subregions. Differences are presented as log_2 fold-change compared with cre-negative mice. *adjusted p<0.05, **adjusted p<0.01, ***adjusted p<0.001, ****adjusted p<0.0001, $^{\#}$p<0.05, $^{\#\#}$p<0.01*

Supplemental Figure 3

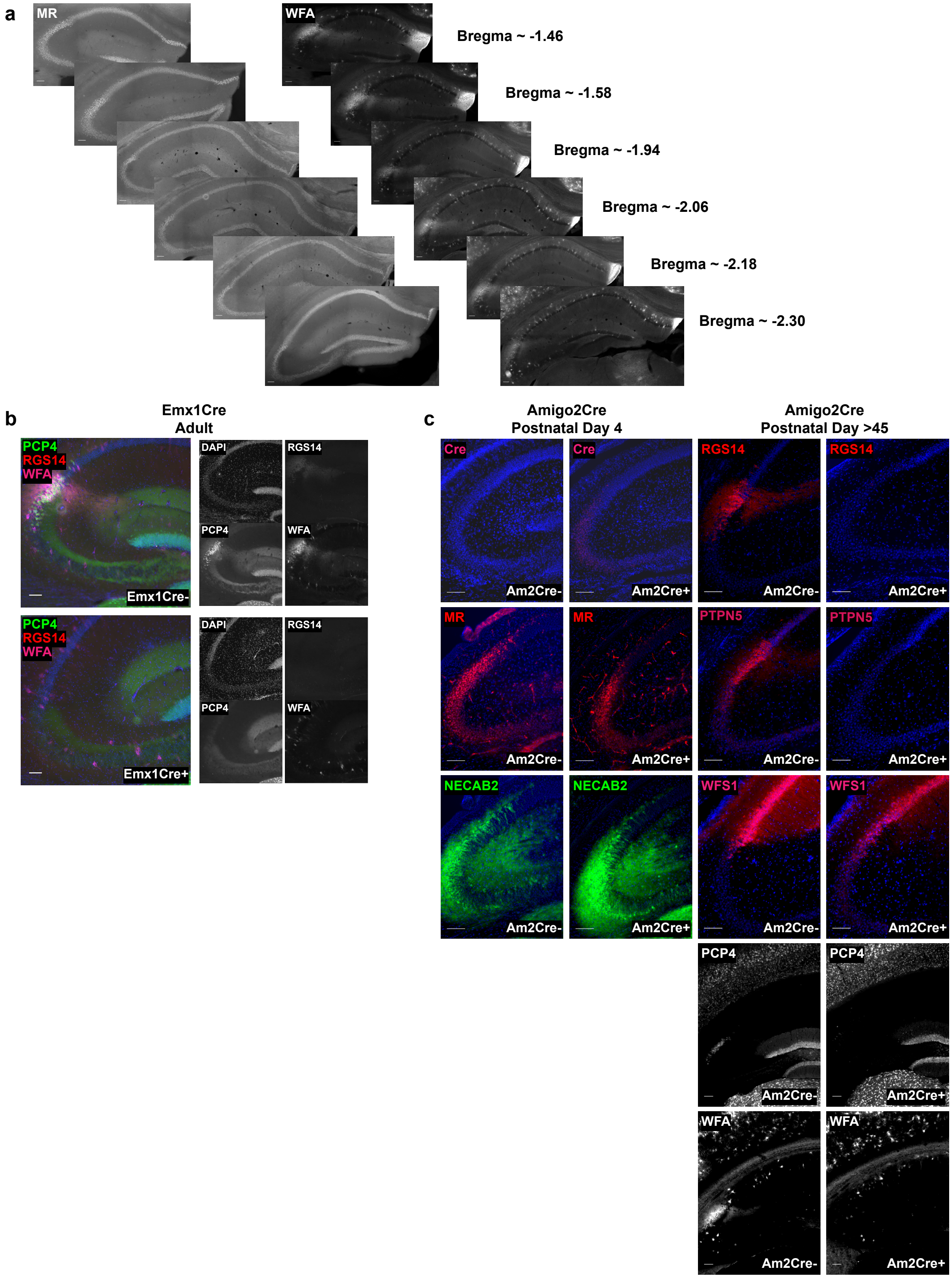

Supplemental Figure 3 Additional images of immunofluorescence staining in Emx1Cre and Am2Cre mice. *Refers to Figures 2, 3, and Supplemental Figures 1,4.* ***(a)*** *Representative coronal sections of the hippocampus showing staining for MR (left) and CA2 marker WFA (right) indicating the rostral to caudal distribution of CA2 markers and the approximate bregma coordinates.* ***(b)*** *Consistent with staining in NesCre+ mice (Figure 2), staining for CA2 markers PCP4 (green), RGS14 (red), and WFA (magenta) was also lost in adult mice with an embryonic knockout of MR using an Emx1 promoter-driven cre recombinase. Individual stains are shown in black and white.* ***(c)*** *Modest*

staining for cre recombinase (red) was detected at PN4 in Am2Cre+ animals; however, neither staining for MR (red) nor for NECAB2 (green) differed in CA2 between Am2Cre- and Am2Cre+ at this age. RGS14 (red) and PTPN5 (magenta) were virtually undetectable in Am2Cre+ mice after PN45, while the CA1 marker WFS1 (magenta) was increased in putative CA2 of Am2Cre+ mice compared with tissue from Am2Cre- mice. CA2 marker PCP4 and perineuronal net marker WFA (black/white) were undetectable in adult cre+ mice. Full statistics are shown in Supplemental Figure 1. DAPI nuclear stain is in blue for all color images. Scale bars = 100µm.

Supplemental Figure 4

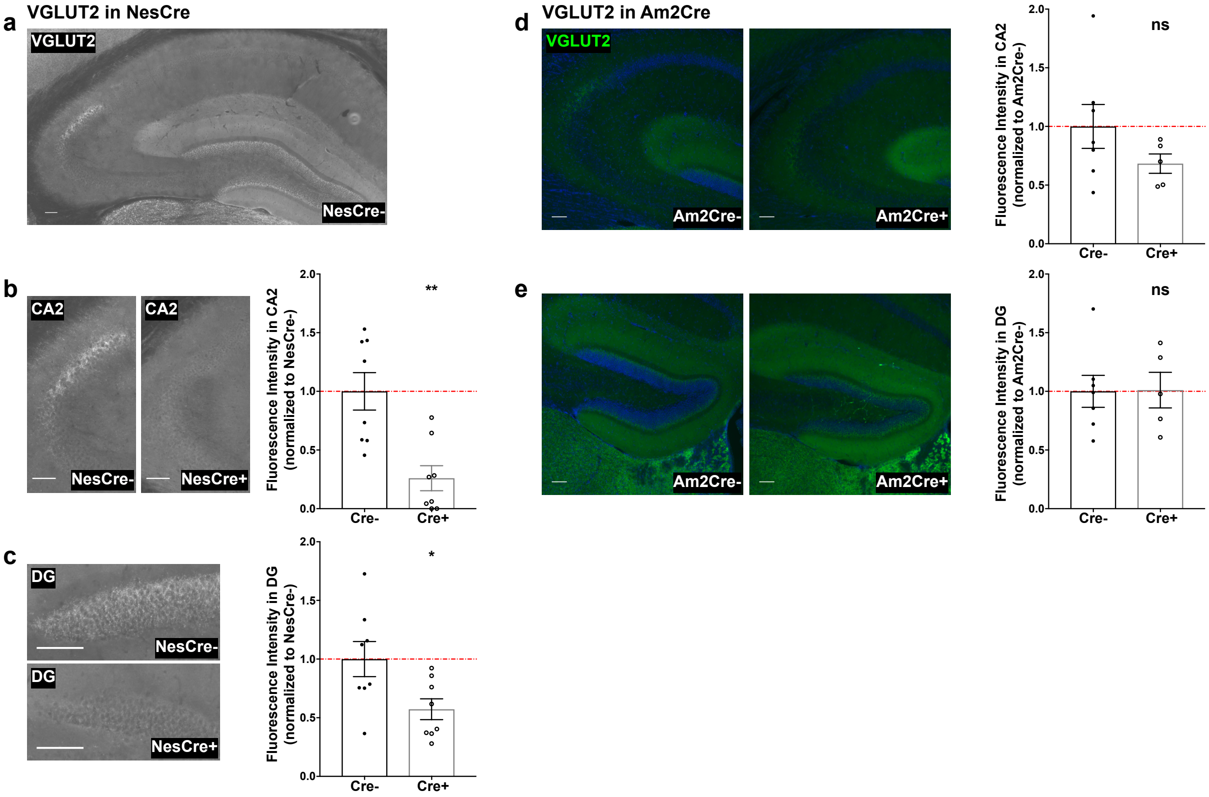

Supplemental Figure 4 Immunofluorescence and quantification of SuM input into CA2. (a) *Representative images of VGLUT2 immunofluorescence in NesCre- mice.* ***(b)*** *VGLUT2 staining in CA2 of NesCre- mice (left) and NesCre+ mice (right). Quantification and analysis showed NesCre+ mice had significantly less VGLUT2 staining than did NesCre- mice (unpaired, two-tailed t-test, t(14)=3.855, p=0.0018).* ***(c)*** *VGLUT2 staining in DG of NesCre- mice (top) and NesCre+ mice (bottom). Quantification and analysis showed NesCre+ mice had significantly less VGLUT2 staining than did NesCre- mice (unpaired, two-tailed t-test, t(14)=2.462, p=0.0274).* ***(d-e)*** *VGLUT2 staining was not significantly different between Am2Cre- mice (left) and Am2Cre+ mice (right) in* ***(d)*** *CA2 (t(10)=1.352, p=0.2061) or in* ***(e)*** *DG (t(10)=0.05062, p=0.9606). Scale bars = 100µm. Refer to Supplemental Figure 3 for bregma coordinates. *p<0.05, **p<0.01,* $^{ns}p>0.05$

Supplemental Figure 5

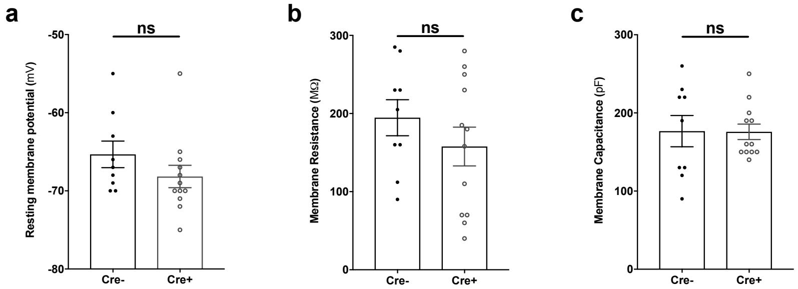

Supplemental Figure 5 Intrinsic properties of NesCre neurons. *Refers to Figure 4. There were no differences in the* ***(a)*** *resting membrane potential (unpaired, two-tailed t-test, t(19)=1.281, p=0.2154),* ***(b)*** *membrane resistance (unpaired, two-tailed t-test, t(19)=1.055, p=0.3048), or* ***(c)*** *membrane capacitance (unpaired, two- tailed t-test, t(19)=0.04034, p=0.9682) between NesCre- (closed circles) and NesCre+ mice (open circles).* $^{ns}p>0.05$

Supplemental Table 1: Data from Social Behavior Testing – Social Investigation

Social Investigation

	**NesCre-**	**NesCre+**	**Am2Cre-**	**Am2Cre+**
**Distance (cm)**	4518 ± 212.8	4439 ± 229	5045 ± 351.6	5292 ± 356.2
**Statistics**	**ns**<br>t(29)=0.2483, p=0.8057		**ns**<br>t(35)=0.4899, p=0.6273	
**Speed (cm/s)**	7.55 ± 0.36	7.4 ± 0.38	8.23 ± 0.55	8.8 ± 0.58
**Statistics**	**ns**<br>t(29)=0.2823, p=0.7797		**ns**<br>t(35)=0.7161, p=0.4787	
**Mean difference in time spent in empty - mouse chambers (s)**	-184.9 ± 34.43<br>****p<0.0001	-124.5 ± 40.52<br>**p=0.0046	-106.1 ± 34.7<br>**p=0.0043	-103.1 ± 37.63<br>**p=0.0096
**Statistics**	**Interaction: ns**<br>F(1,29)=1.29, p=0.2653<br>**Genotype: ***<br>F(1,29)=4.511, p=0.0423<br>**Chamber: ******<br>F(1,29)=33.87, p<0.0001		**Interaction: ns**<br>F(1,35)=0.0034, p=0.954<br>**Genotype: ns**<br>F(1,35)=2.386, p=0.1314<br>**Chamber: *****<br>F(1,35)=16.7, p=0.0002	

Supplemental Table 2: Data from Social Behavior Testing – Social Discrimination

Social Discrimination	NesCre-	NesCre+	Am2Cre-	Am2Cre+
**Distance (cm)**	4489 ± 197.1	4102 ± 240.6	4984 ± 254.5	5421 ± 409.5
**Statistics**	**ns** t(29)=1.249, p=0.2216		**ns** t(35)=0.9352, p=0.3561	
**Speed (cm/s)**	7.49 ± 0.33	6.84 ± 0.4	8.26 ± 0.4	8.84 ± 0.69
**Statistics**	**ns** t(29)=1.262, p=0.2169		**ns** t(35)=0.7425, p=0.4627	
**Time spent in either mouse chamber (s)**	420.9 ± 27.03	350.5 ± 24.41	371.4 ± 14.86	387.4 ± 13.77
**Statistics**	t(29)=1.852, p=0.0742		t(35)=0.7798, p=0.4407	
**Mean difference in time spent in familiar - novel chambers (s)**	-96.51 ± 39.33<br>*p=0.0204	-75.62 ± 46.28<br>p=0.1131	-73.61 ± 32.81<br>*p=0.0313	-13.22 ± 35.59<br>p=0.7126
**Statistics**	**Interaction: ns**<br>F(1,29)=0.1183, p=0.7334<br>**Genotype: ns**<br>F(1,29)=3.43, p=0.0742<br>**Chamber: ****<br>F(1,29)=8.03, p=0.0083		**Interaction: ns**<br>F(1,35)=1.556, p=0.2205<br>**Genotype: ns**<br>F(1,35)=0.6081, p=0.4407<br>**Chamber: ns**<br>F(1,35)=3.217, p=0.0815	

Supplemental Tables 3: Data from Open Field and Novel Object Behavior Testing

Open Field (Day 1)

	NesCre-	NesCre+	Am2Cre-	Am2Cre+
**Distance (cm)**	1814 ± 97.94	2942 ± 244.5^{s}	1525 ± 94.06	1861 ± 160.2
**Statistics**	**** t(29)=4.763, p<0.0001		**ns** t(35)=1.872, p=0.0696	
**Speed (cm/s)**	3.07 ± 0.17	5.1 ± 0.41^{s}	2.58 ± 0.17	3.15 ± 0.27
**Statistics**	*** t(16.11)=4.627^{+}, p=0.0003		**ns** t(35)=1.861, p=0.0712	
**Time in center (s)**	265.8 ± 28.38	176.7 ± 21.72	258.2 ± 26.21	242.9 ± 20.67
**Statistics**	* t(29)=2.33, p=0.027		**ns** t(35)=0.4453, p=0.6588	

^{s} *Post hoc* analysis revealed a sex difference within this group: Males higher than females
$^{+}$Welch's correction applied to account for unequal variances between groups

Habituation (Day 2)

	NesCre-	NesCre+	Am2Cre-	Am2Cre+
**Distance (cm)**	1331 ± 70.06	2203 ± 326.1	1295 ± 76.37	1248 ± 110.8
**Statistics**	* t(13.11)=2.615^{+}, p=0.0213		**ns** t(35)=0.3636, p=0.7184	
**Speed (cm/s)**	2.23 ± 0.12	3.71 ± 0.54	2.2 ± 0.13	2.09 ± 0.19
**Statistics**	* t(13.18)=2.649^{+}, p=0.0199		**ns** t(35)=0.5045, p=0.6171	
**Time in center (s)**	185.5 ± 28.9	145.5 ± 30.42	133.2 ± 13.59	152.4 ± 24.74
**Statistics**	**ns** t(29)=0.9369, p=0.3565		**ns** t(25.19)=0.6803^{+}, p=0.5025	

$^{+}$Welch's correction applied to account for unequal variances between groups

Novel Object Exposure (Day 3)

	NesCre-	NesCre+	Am2Cre-	Am2Cre+
**Distance (cm)**	1614 ± 122	2306 ± 275.3	1693 ± 112.2	1355 ± 115.5
**Statistics**	* t(16.72)=2.299^{+}, p=0.0347		* t(35)=2.091, p=0.0439	
**Speed (cm/s)**	2.88 ± 0.23	3.96 ± 0.45	2.88 ± 0.19	2.37 ± 0.18
**Statistics**	* t(29)=2.317, p=0.0278		**ns** t(35)=1.94, p=0.0605	
**Time in investigation zone (s)**	37.7 ± 4.6	69.16 ± 6.22	41.43 ± 5.14	76.83 ± 13.82
**Statistics**	*** t(29)=4.165, p=0.0003		* t(20.39)=2.401^{+}, p=0.026	

$^{+}$Welch's correction applied to account for unequal variances between groups

Supplemental Table 4: Data from Elevated Plus Maze Behavior Testing

Elevated Plus Maze

	**NesCre-**	**NesCre+**	**Am2Cre-**	**Am2Cre+**
**Distance (cm)**	1190 ± 90.2	1185 ± 97.25[s]	1380 ± 69.34[s]	1128 ± 119.9
**Statistics**	**ns** t(29)=0.03461, p=0.9726		**ns** t(35)=1.887, p=0.0674	
**Speed (cm/s)**	4 ± 0.3	4.05 ± 0.32[s]	4.63 ± 0.23	3.77 ± 0.4
**Statistics**	**ns** t(29)=0.1036, p=0.9182		**ns** t(35)=1.92, p=0.063	
**Open Arm Entries**	12.06 ± 0.98	12.23 ± 1.19	11.9 ± 0.86	10 ± 1.44
**Statistics**	**ns** t(29)=0.1142, p=0.9099		**ns** t(35)=1.174, p=0.2482	
**Open Arm Duration (s)**	132.5 ± 16.42	143.5 ± 17.15	115.6 ± 11.04	181.2 ± 19.57
**Statistics**	**ns** t(29)=0.4535, p=0.6535		** t(25.62)=2.919[+], p=0.0072	

[+]Welch's correction applied to account for unequal variances between groups
[s] *Post hoc* analysis revealed a sex difference within these groups: Females higher than males

Supplemental Table 5: Antibodies used in immunofluorescence experiments

Antibody	Manufacturer	Catalog Number	Dilution
MR	Dr. Celso E. Gomez-Sanchez	6G1	1:100
GR	Cell Signaling Technology	3660S	1:250
NECAB2	Novus	Nbp1-84002	1:500
WFS1	Protein Tech	11558-I-AP	1:250
WFA	Vector	B-1355	1:500
PCP4	Santa Cruz	Sc-74816	1:500
RGS14 (N133/21; RRID: AB_2179931)	UC Davis/NIH Neuromab Facility	75-170	1:500
STEP/PTPN5	Cell Signaling Technology	Mab #4396	1:500
VGLUT2	Synaptic Systems	135 421	1:200
Cre	Synaptic Systems	257 003	1:500
Goat anti Mouse IgG (H+L) Highly Cross Adsorbed Secondary Antibody, Alexa Fluor® 488	Invitrogen	A11001	1:500
Goat anti Mouse IgG (H+L) Highly Cross Adsorbed Secondary Antibody, Alexa Fluor® 568	Invitrogen	A11004	1:500
Goat anti Mouse IgG (H+L) Highly Cross Adsorbed Secondary Antibody, Alexa Fluor® 633	Invitrogen	A21052	1:500
Goat anti-Rabbit IgG (H+L) Highly Cross Adsorbed Secondary Antibody, Alexa Fluor® 488	Invitrogen	A11034	1:500
Goat anti-Rabbit IgG (H+L) Highly Cross Adsorbed Secondary Antibody, Alexa Fluor® 568	Invitrogen	A110036	1:500
Goat anti-Rabbit IgG (H+L) Highly Cross Adsorbed Secondary Antibody, Alexa Fluor® 633	Invitrogen	A21071	1:500

Supplemental Table 6: Custom codeset for NanoString analysis (page 1 of 3)

*Genes that did not quantify above noise are designated in the Notes column. These genes were not included in analysis. HK = housekeeping gene

Gene	Accession #	Position	Target Sequence	Target	Notes
Acan	NM_007424.2	4861-4960	CAGAACTTTGGTGGAATCTATAACTCAGGCTCCTACTGCTCAAGAAGCTGGAGAAGGACCTTCGGGCATTTTGGAATTCAGTGGTGCCCATTCTGGGACA	CA2	
Actb	NM_007393.3	72-171	AGTTCGCCATGGATGACGATATCGCTGCGCTGGTCGTCGACAACGGCTCCGGCATGTGCAAAGCCGGCTTCGCGGGCGACGATGCTCCCCGGGCTGTATT	HK	
Adcyap1r1	NM_001025372.1	5811-5910	TGGGACACTCCTCAAGTGAGCCAAATGCCCGTTCTTTCTCCCTTCCTTGAACTCCAGGTCCTGTGCTGTGTCTGTACTCAGCCATGTGTGTTTTGTCTGC	DG	
Aif1	NM_019467.2	56-155	CTGGAGCAGCCTGCAGACTTCATCCTCTCTCTTCCATCCCGGGGAAAGTCAGCCAGTCCTCCTCAGCTGCCTGTCTTAACCTGCATCATGAAGCCTGAGG	OTHER	*
Akap7	NM_018747.4	1836-1935	CTTAATTAATGAAGCTTTGCACCGAGAAAGGATGGAGCTGAAATCCAAAGTGAAACAGATAAAAGAACTTTTGTTAAAGCCTGAGACTCAGGCCAAGATT	DG	
Alas1	NM_020559.2	1035-1134	ACTAGGAATATTTCTGGAACGAGCAAGTTCCACGTAGAACTGGAGCAGGCACTGGCCGACCTCCACGGCAAAGATGCGGCGCTCTTGTTCTCTTCCTGTT	HK	
Amigo2	NM_178114.4	2426-2525	AATGGTGTCTTTTTAAGATGGTTGCTAGGAAGTGAGGCAGAGCACACTGGTTGCTTCTTGGACTGGGTCAGGAGACGGTTGAGTTTGAGAGTTTTGAGTA	CA2	
Apoe	NM_001305844.1	904-1003	CCTGCAGGCGGAGATCTTCCAGGCCCGCCTCAAGGGCTGGTTCGAGCCAATAGTGGAAGACATGCATCGCCAGTGGGCAAACCTGATGGAGAAGATACAG	OTHER	
Ar	NM_013476.3	2216-2315	CATGGGTTGGCGGTCCTTCACTAATGTCAACTCCAGGATGCTCTACTTTGCACCTGACTTGGTTTTCAATGAGTACCGCATGCACAAGTCTCGGATGTAC	OTHER	
Avpr1b	NM_011924.2	3333-3432	AGCCTGGATGTCTCAGGTATAAGAGTCAAAGTGGGTATACATGTGGTTTGCAATCATTGCAGATCTACAAGCTTTGGAGCAAAATTCCTGGGAACAGCCT	CA2	*
Bdnf	NM_007540.4	3261-3360	AGTCCCGTCTGTACTTTACCCTTTGGGGTTAGAAGTCAAGTTGGAAGCCTGAATGAATGGACCCAATGAGAACTAGTGTTAAGCCCATTTCCCTAGTCAG	OTHER	
Boc	NM_172506.2	3791-3890	AACGTGGGATGCGGTTCTCGCCTAGCCCATCAGTTCATGTGTCCTTTGAAACACCACCTCCCACAATTTAGGCCGAAGCTAATATCCCAGAAAGACTATA	CA3	
Bok	NM_016778.2	636-735	CCCTGGTTGACTGCCTGGGGGAGTTTGTACGCAAGACCTTGGCTACCTGGCTTCGGAGGCGTGGTGGATGGACGGATGTCCTCAAGTGTGTGGTCAGCAC	CA3	
Cacng5	NM_001199301.1	695-794	GCAGAGACCTATTTCAACTACAAGTATGGATGGTCATTTGCCTTTGCTGCCATCTCCTTCCTTTTAACAGAGAGTGCTGGGGTGATGTCTGTGTACCTGT	CA2	
Tex40	NM_001039494.2	361-460	TGGAGCATGGGGAGGACCACGAAGAATCTGAGACCTCCTTAGCATCGTCTACTTCAGAATCACTAATATTCAGCCTATGGAAGCCACATCGAACCTACTG	CA2/CA3	
Cdh9	NM_009869.1	2341-2440	GGGGGTAACGACAGTGATCTGAACTGAATCCTATATGACTTCATCAACATTAGTAGAAACAGTATCTTCAATACCAGATTGAGTGGCCTGTGTTGTCCCT	CA3/DG	
Cdon	NM_021339.2	115-214	ATTTTATTTCTGAGCCACTCTCTGCTGTCCAGAAGCTTGGTAGACCCGTGGTCCTACATTGTTCTGCTAAACCTGTTACTGCCCGAATCTCATGGTTGCA	DG	
Cntn4	NM_001109751.1	591-690	GTTGCCATGGGAACTGCTAGTGCTGCAATCATTCATGTTGTGCCTTGCAGATGACTACACACTGCATGGCCCAGTTTTTGTTCAAGAACCAAGTCATGTC	CA1	
Crhbp	NM_198408.3	1111-1210	TAGTCTGTGAATGGCCGGCTTCAGTGATTGACATGCTGTTAGCCCAGTGAGTTCTCCACAGTTACTGTATGTGATCTTCATGGGCAGCAAATGGAAAGTC	OTHER	
Crhr1	NM_007762.4	1749-1848	GTTGGGGAGGCCAACTGTTACCCTGGGGCATCATGGAAAACTCCCTTCTGAGACTGTAAAGTCTCTGAGTGTTAGCGATGCCTTGGGATGCTACCGAGGA	OTHER	
Ctsc	NM_009982.4	287-386	AACAGAAGAAAAGGTAGTGGTACACCTTAAGAAGTTGGATACTGCCTACGACGAGCTGGGCAATTCCGGGCATTTTACCCTCATTTACAACCAAGGCTTC	CA2	
Dcx	NM_010025.2	8576-8675	GAGTGATACAAAGGCAGTGTTTCAACAAGACACTCTCTTCTTTGGTTTGCTGTTCTCTCTTGGACTGGAATGTTTGGCAAGGCCCATGTAAACTGGTTAG	OTHER	
Dicer1	NM_148948.2	1391-1490	TCGAGATCCTGCGCAAATACAAGCCCTATGAGCGACAGCAGTTTGAAAGTGTTGAGTGGTACAATAACAGGAATCAGGATAATTACGTGTCCTGGAGTGA	OTHER	
Dock10	NM_175291.3	1957-2056	GATATTGCTGTGGACAACATTCCCTTGGAGCACCCAAACTGTGTAACATCGTCCTTTATCCCTGTCAAGCCCTTCAACGTGTCGGCTCAGTCAGAACCTA	DG	
Enox2	NM_001271448.1	547-646	GCCTGAAAATGGGACAGAGCAGATCATTGTGGAAGTGTTTGAACAGTGTGGAGAGATTATTGCTATCCGGAAGAGCAAAAAGAACTTCTGTCACATTCGC	CA3	
Fgf2	NM_008006.2	288-387	CTCTACTGCAAGAACGGCGGCTTCTTCCTGCGCATCCATCCCGACGGCCGCGTGGATGGCGTCCGCGAGAAGAGCGACCCACACGTCAAACTACAACTCC	CA2	*
Fhod3	NM_175276.3	3876-3975	ACTCTGGGCTTCATCCTGTCTACTCTCCTTGCCATTGGGAACTTTCTAAATGGAACTAATGCCAAAGCATTTGAGTTAAGCTACCTCGAGAAGGTTCCAG	CA2	
Fkbp5	NM_010220.3	2126-2225	ACAGACTCTGTTGTTCCTCCAACGCCCGTCATTAGTGACAGCTTTCTCTCTGAGTTTCTGTGGTGTGGAGAGTGGGTAGAAGTAGGTTTATCTTTCCCGC	OTHER	
Fnbp1l	NM_001114665.2	959-1058	GGTAGTAGACTCCTTCAAGTCTGGCTTTGAACCTCCAGGAGACTTTCCATTTGAAGATTACAGTCAGCACATTTACAGAACCATTTCTGACGGGACCATC	CA1	
Frmd4b	NM_145148.2	2556-2655	AAGCCATTTTTCACCCTCTCCAAATCCCAAAGAAGCAGCAGCACGGAGATCCTTGACGACGGGTCTTCGTATACCAGCCAGTCAAGCTCCGAGTATTACT	DG	
Fxyd1	NM_052991.4	205-304	ATCGGCGGCCTCACTATCGCTGGGATCCTCTTCATCTTGGGCATCCTTATCATCCTTAGCAAGAGATGTCGATGCAAATTCAACCAACAGCAGAGAACTG	CA3	
Gad1	NM_008077.4	747-846	AGAGACACCCTGAAGTACGGGGTTCGCACAGGTCACCCTCGATTTTTCAACCAGCTCTCTACTGGTTTGGATATCATTGGTTTAGCTGGTGAATGGCTGA	DG	
Gad2	NM_008078.2	770-869	ATAATTGGGAATTGGCAGACCAACCGCAAAATCTGGAGGAAATTTTGACGCATTGCCAAACAACTCTAAAATATGCAATTAAAACAGGGCATCCCCGATA	OTHER	
Galntl6	NM_175032.3	2515-2614	AGGGTACATGGCGTCGAACACTTACATTACATGCTCTCATCCAAAGGCCATGCTATCCGTCATGGTTCATAAGAGCTCATGGTCCACCAATTAGCTGAAG	CA1	
Gapdh	NM_008084.2	216-315	GGCACAGTCAAGGCCGAGAATGGGAAGCTTGTCATCAACGGGAAGCCCATCACCATCTTCCAGGAGCGAGACCCCACTAACATCAAATGGGGTGAGGCCG	HK	
Gfap	NM_001131020.1	611-710	CACCCTGGCTCGTGTGGATTTGGAGAGAAAGGTTGAATCGCTGGAGGAGGAGATCCAGTTCTTAAGGAAGATCTATGAGGAGGAAGTTCGAGAACTCCGG	OTHER	
Gria1	NM_001252403.1	2477-2576	ATCCTGATTGGAGGGCTGGGATTGGCCATGCTGGTTGCCTTAATCGAGTTCTGCTACAAATCCCGTAGCGAGTCGAAGCGGATGAAGGGTTTCTGTTTGA	OTHER	
Gria2	NM_001039195.1	301-400	GACCTCAAAATGCAGAGGATCTAATTTGCTGAGGAAAACGGTCAAAGAAGGAAAAGGAGGAAAGGGAAACGAGGGGATATTTTGTGGATGCTCTACTTTT	OTHER	
Gria3	NM_016886.3	391-490	GCTTTCCGCTTTGCTGTGCAGTTATACAACACCAACCAGAACACCACTGAGAAGCCCTTCCATTTGAACTACCACGTAGACCACTTGGATTCCTCCAATA	OTHER	
Gria4	NM_001113180.1	1275-1374	GATTTTAATACGCCCATGGTGACGAAACTAATGGATCGCTGGAAGAAACTAGATCAACGAGAATATCCAGGATCTGAAACACCTCCAAAGTACACTTCTG	OTHER	
Grik1	NM_010348.3	1336-1435	GGACTTGTTTGCTTTGGATCTGGAACTCTACAGGTACAGTGGTGTAAATATGACTGGATTTCGGTTGCTGAATATTGACAACCCTCACGTGTCATCCATC	OTHER	
Grik2	NM_010349.2	257-356	CTAATGGGTTTGGGAAGCGGAGACTCCTTCCTCTTTCTGTGACCATGCCGTGATTGTGTTTGCGGCCACTATTCCACGCATCCTTCTTCTCGTCCAAGCC	OTHER	
Grik3	NM_001081097.2	6365-6464	TCTCTTATGGACAGTCACTGATGAAATTTGAGCACTTTGTGGGGGCTCAGGTGGCTCCTGGGCAGTTGTGTTGGCCAGTGAGTTGGACTTAGAATCCTTG	OTHER	
Grik4	NM_175481.5	959-1058	GAGAATGGACAGCCTTGTGGATGATAGAGTCAACATCTTAGGATTTTCCATTTTCAACCAATCCCATGCATTCTTCCAAGAGTTCTCCCAGAGCCTCAAC	CA2/CA3	
Grik5	NM_008168.2	2773-2872	GTTTGGGCATGGAGAACATTGGCGGCATTTTTGTCGTGCTGATCTGTGGCCTCATCATTGCTGTCTTCGTGGCGGTCATGGAGTTCATCTGGTCCACGCG	OTHER	
Grin1	NM_008169.2	493-592	ACAGATGGCCCTGTCAGTGTGTGAGGACCTCATCTCTAGCCAGGTCTACGCTATCCTAGTTAGTCACCCGCCTACTCCCAACGACCACTTCACTCCCACC	OTHER	
Grin2a	NM_008170.2	1789-1888	CCTTCTTTTACCATTGGAAAAGCTATATGGCTCCTCTGGGGCCTGGTCTTCAACAATTCTGTGCCCGTCCAGAATCCTAAAGGCACAACCAGCAAGATAA	OTHER	
Grin2b	NM_008171.3	6341-6440	GGGAAAGCTCTTCGTATAAGGCTTTGTGAAAGAGCCATTACAGTAGGGTGAGAGAGGGGGATGTTTTTAGTCATTAACGGTAGGGTTAGTGAGAAAGGGG	OTHER	
Grin2c	NM_010350.2	2409-2508	GCTTTTCTGGAGCCCTACAGCCCTGCCGTGTGGGTGATGATGTTTGTAATGTGCCTCACGGTGGTTGCCATCACTGTCTTCATGTTCGAGTATTTCAGCC	OTHER	
Grin2d	NM_008172.2	1202-1301	ACATCACCTGGGATAACCGAGACTACTCCTTCAATGAGGATGGCTTTCTGGTAAACCCGTCACTGGTAGTCATCTCCCTCACCAGAGACAGGACGTGGGA	OTHER	
Grin3a	NM_001033351.1	1333-1432	CTCAGTGGTTCCATCAAAGTAAAGGGATCCACCATCGTCAGCTCAGAAAACAACTTTTTCATCTGGAACTTGCAGTATGACCCTATGGGAAAGCCAATGT	OTHER	
Grm1	NM_001114333.2	2126-2225	CACAGGCTGTGAGCCCATTACTATCCGTTACCTCGAGTGGAGTGACATAGAATCCATCATAGCCATCGCCTTTTCTTGCCTGGGCATCCTCGTGACGCTA	OTHER	
Grm2	NM_001160353.1	2771-2870	GGATCCCAGCTTGTTCCTACTGTTTGCAATGGCCGTGAGGTGGTAGACTCAACAACATCGTCGCTTTGAAGATCCCACACTCCTGCCCTGACATGGCTGC	DG	
Grm3	NM_181850.2	2526-2625	AGAAGCGGGAAACAGTCATCCTAAAATGCAATGTCAAAGATTCCAGCATGTTGATCTCTCTGACCTATGACGTGGTTCTGGTGATTCTATGCACTGTGTA	OTHER	
Grm4	NM_001013385.1	823-922	TAGCTACGGCGAGAGCGGCGTGGAGGCCTTTATCCAGAAGTCCCGAGAGAACGGAGGCGTGTGCATTGCCCAGTCGGTGAAGATTCCACGGGAACCCAA	OTHER	
Grm5	NM_001143834.1	4243-4342	CTCCGAATCGGCCCTCTGCATCCCATCCTCTCCCAAATATGACACTCTCATCATCAGAGATTACACGCAGAGTTCTTCATCGTTGTGAGCCACTGGAAAC	OTHER	
Grm7	NM_177328.3	1296-1395	TGTGGAAGGATTTGATGCTTACTTCACATCCCGGACACTTGAAAACAACAGGAGAAATGTATGGTTTGCCGAATACTGGGAAGAAAACTTCAACTGCAAG	OTHER	

Supplemental Table 6: Custom codeset for NanoString analysis (page 2 of 3)

*Genes that did not quantify above noise are designated in the Notes column. These genes were not included in analysis. HK = housekeeping gene

Gene	Accession #	Position	Target Sequence	Target	Notes
Grm8	NM_008174.2	1771-1870	GCTCCGGGACGCTACGATATCTTCCAATATCAGATAAACAACAAAAGTACAGAATACAAAATCATCGGCCACTGGACCAATCAACTTCACCTAAAAGTGG	OTHER	
Gsto1	NM_010362.2	566-665	GGCATGGATAATTACAAGAGCTTCCTTGGTGGGGATTCTCCTTCTATGGTTGATTATCTTACTTGGCCTTGGTTTCAGCGACTGGAAGCATTGGAGCTCA	CA2	
Hprt	NM_013556.2	31-130	TGCTGAGGCGGCGAGGGAGAGCGTTGGGCTTACCTCACTGCTTTCCGGAGCGGTAGCACCTCCTCCGCCGGCTTCCTCCTCAGACCGCTTTTTGCCGCGA	HK	
Hs6st2	NM_001077202.1	1431-1530	GCGCAAGACCCAGTACCTGTTTGAGAAGACCTTCAACATGAACTTTATCTCGCCGTTTACCCAGTATAATACCACTAGGGCCTCTAGTGTTGAGATCAAT	CA1	
Htr1a	NM_008308.4	3171-3270	CACACAAATTTCGCAGATCTCTGGGCTCTCATTGTGAAGGGTATCAACACCTCACCATTACCTACCTAGGATCTTGATCTCAGAGACCTAAAAGAGAGAG	OTHER	
Htr1b	NM_010482.1	626-725	CGGTGGGCGCTTTCTATTTACCCACCCTGCTCCTCATCGCCCTCTATGGCCGCATCTATGTGGAAGCCCGCTCTCGGATTTTGAAACAGACACCCAACAA	OTHER	
Igfbp5	NM_010518.2	2771-2870	CTTCCAACCCGCAGAAAGTAACTGTCTCACACCACACCACATAAACCTGCCAGATCCATCTGTAACCCACTGGCCTGCCCAGACCTTTTTTTCCCATCTG	DG	
Igsf3	NM_207205.2	6267-6366	CACATCAGAGGGACCAAATTGGCTCAGTCACGAAGAAAGAATGTCTTTTGTTCTGTTCCTTCGATTCATGGGTGTGGGCTCTTGGTGGGTAGTTGCTGAC	DG	
Il1rap	NM_134103.2	946-1045	GAGAAAGAACCAGGAGAGGAACTGGTTATTCCCTGCAAAGTCTATTTCAGTTTCATTATGGACTCCCACAATGAGGTCTGGTGGACCATTGATGGAAAGA	DG	
Iyd	NM_027391.3	271-370	GGTCCCAGGAATTCTATGAGCTCCTCAATAAGAGACGCTCCGTCAGGTTTATCAGCAGCGAGCACGTCCCAATGGAAGTCATTGAAAATGTCATCAAAGC	CA2/CA3	
Klhl13	NM_026167.3	1937-2036	GGTGGCTATTCATGGAATAATCGCTGTATGGTAGAGATAGTGCAGAAGTATGATCCAGAGAAAAATGAATGGCATAAGGTTTTCGATCTGCCAGAGTCTC	CA1	
Ldb2	NM_001077398.1	129-228	CTTTGTACTGTGAAGAACACAGCCCATGTGCTCTGCATGGACGTTCCTGATACTCTATTAAGCTTGATTTTCGACAAGCAGGCAAGATGTCCAGCACACC	CA1	
Lrrtm3	NM_178678.4	356-455	TGGAGTGTTCTGCATGGCTGGTAAAGAATAATGTTCCAAAATCGGTCCATCTCCCAAGGGGTCCAATTTTTCTTCCTGGGTGTCAGCGAGCCCTGACTCA	DG	
Map3k15	NM_001163085.2	2101-2200	GAAATAGCTCTGCACAAGTATCTCAAACATCGCAACATCGTCCAGTACCTAGGCTCTGTTTCAGAGAATGGCTACATTAAGATATTTATGGAGCAGGTGC	CA2	*
Mecp2	NM_010788.2	756-855	AAAGGGAGCGGCACTGGGAGACCAAAGGCAGCAGCATCAGAAGGTGTTCAGGTGAAAAGGGTCCTGGAGAAGAGCCCTGGGAAACTTGTTGTCAAGATG	OTHER	
Mef2c	NM_001170537.1	4342-4441	TTCTACTACTAAAGGTATCAATGGAACATGAAGACGAGTATTTAGGCAGAAGCAAAACAGGAAACCATCCTTACAAACATGCTTACCTGCACATCTGTTT	DG	
Mgat4c	NM_026243.4	931-1030	AGTCATAGCATCCTTGGAAGGAACATACTGGGTAACTCTTGAGTTCTCTAAACTTGGCTACATTGGTAAACTCTATCATTCTCACGATCTCCCACGTCTG	CA3/CA2	
Ndst3	NM_031186.2	1433-1532	TCTGAAACCAGCTAGATATCGGAGAGGCTTCATTCACAAAAACATCATGGTTCTCCCAAGACAAACCTGTGGGCTCTTCACCCACACAATTTTCTACAAG	CA1	
Necab2	NM_054095.2	787-886	ATGGGCTACACTAAGAAGGTGTATGAAGGTGGGAGCAATGTGGACCAGTTTGTGACACGCTTCCTTCTGAAGGAGACAGCCAATCAGATCCAGTCGCTGC	CA2	
Nectin3	NM_021495.4	635-734	TGCAAAGCCGTTACATTCCCACTTGGAAATGCTCAGTCCTCTACAACAGTGACTGTGTTAGTTGAACCCACAGTGAGCCTGATAAAAGGGCCGGATTCTT	CA2/CA3	
Nos1	NM_008712.2	2986-3085	GCTGGAAGAGGAATAAATTCCGCCTCACTTATGTGGCAGAAGCTCCAGAGCTGACCCAAGGTCTTTCCAATGTTCACAAAAAGCGAGTCTCAGCCGCCCG	CA1/CA3	
Npy	NM_023456.2	231-330	GACACTACATCAATCTCATCACCAGACAGAGATATGGCAAGAGATCCAGCCCTGAGACACTGATTTCAGACCTCTTAATGAAGGAAAGCACAGAAAACGC	OTHER	
Nr3c1	NM_008173.3	1801-1900	ACCAGGATTCAGAAACTTACACCTGGATGACCAAATGACCCTTCTACAGTACTCATGGATGTTTCTCATGGCATTTGCCCTGGGTTGGAGATCATACAGA	OTHER	
Nr3c2	NM_001083906.1	3461-3560	ATACAGATTCCTGGTATGTAATAAACAGAACACGTGGCGTGGAACTGTGGCCTTCCAGGCACGTTTTTCTGCATCGCTGGCATACACACTCGTTAGTGTC	OTHER	
Ntf3	NM_008742.2	306-405	CAATTCCCTCATCATCAAGCTGATCCAGGCGGATATCTTGAAAAACAAGCTTTCCAAACAGATGGTGGATGTTAAGGAAAATTACCAGAGCACCCTGCCC	CA2/DG	
Ntrk2	NM_001025074.1	1346-1445	AAGATCAAGATTCTGTGAACCTCACTGTGCATTTTGCGCCAACTATCACGTTTCTCGAGTCTCCAACCTCAGATCACCACTGGTGCATTCCATTCACTGT	OTHER	
Oaz1	NM_008753.4	993-1092	GCTATAGCAGTCTCCTTTGAAGTCTGGAAAAATAGTGTCACCTCCCCTGGCTCAAATCCAATAAAGTGATCTCGTTCATTGGCAAAAAAAAAAAAAAAAA	HK	
Oxtr	NM_001081147.1	3195-3294	CTGTGCACTCTGGAGTCCCTTTGAAATACCCAGACTTAATTCTGGACTTACTGAAATATATCAAGGGCGCTTCCAAAAAGCCTAGAATCTGTAGATCCAC	OTHER	
Pantr1	NR_027826.1	162-261	TCCTGAGTCAACATGGCCGTTCCTAACTTTGAAGAGACTTTAGACATTCTTTGTTACAATGAACCTAGAAGAAAGAAGAGAAATATTACAGCGACTGTGC	CA1	
Pcp4	NM_008791.2	377-476	CTTCCTGGTCCACCTGAAGACACCAAGTTCAACCACCATCCATCAAGAAATGAAAAGAACAATACCCTAGAGAGAAGTCATCCTCACTCAAGACACACCC	CA2/DG	
Polr1b	NM_009086.2	2796-2895	TGCCTTTCACTGAGAGTGGCATGATGCCGGACATTCTGTTTAATCCTCACGGGTTTCCCTCCCGTATGACCATAGGTATGTTAATCGAGAGCATGGCTGG	HK	
Pou3f2	NM_008899.2	1742-1841	GGAAAGTAAAACACTGGACTATCCTATATCAGGTAGCAGGTGTAATAATGGTTTTTTGACCTTTGCAGGCGAGAGTACCCAGGCAATGAAGTAGAATGAA	CA1	
Ppia	NM_008907.1	391-490	CCAAGACTGAATGGCTGGATGGCAAGCATGTGGTCTTTGGGAAGGTGAAAGAAGGCATGAACATTGTGGAAGCCATGGAGCGTTTTGGGTCCAGGAATGG	HK	
Prkcb	NM_008855.2	8333-8432	CAGAGTCCATTGCCGAGCCTGTGTGTAAGGCTTCAGGCCGAATGTCTGTTACCAATTCCAGTCTTCCAGGATTCATGGTGCCTCTATTGGCATCCGTCAT	CA1/CA3	
Prkcd	NM_011103.2	1266-1365	AGGACCACCTGTTCTTCGTGATGGAGTTTCTCAATGGGGGTGACCTGATGTTCCACATTCAGGACAAAGGCCGCTTCGAACTCTACCGGGCTACGTTTTA	CA3	
Prss23	NM_029614.3	531-630	GGCAGATTTATGGCTACGATGGCAGGTTTAGCATTTTTGGGAAGGACTTCCTGCTCAATTATCCTTTCTCAACATCGGTGAAGTTGTCTACTGGCTGCAC	CA2	
Ptgs1	NM_008969.3	1271-1370	CATCGCCATGGAATTTAACCATCTCTATCACTGGCATCCACTCATGCCCAACTCCTTCCAAGTGGGCTCACAAGAGTACAGCTACGAGCAGTTTTTATTT	CA2	
Ptpn5	NM_001163565.1	2771-2870	TCTTTCTGTGTCACTATGGGAAACCGCAAGTGTCTCCCTACATGTTGCTCTGTGTGTGTTCCCTTGTCCATGCGTGTGTTGAGAGCCCATGAACAGGGCA	CA2	
Pvalb	NM_013645.3	61-160	GAAGGCGATAGGAGCCTTTGCTGCTGCAGACTCCTTCGACCACAAAAAGTTCTTCCAGATGGTGGGCCTGAAGAAAAAGAACCCGGATGAGGTGAAGAAG	OTHER	
Qpct	NM_027455.2	633-732	CATCATGCAACGAATTCAGAGACTTCAGGCTGAGTGGGTCGTGGAAGTTGACACCTTCCTGAGTAGGACTCCCTATGGCTATCGGTCCTTCTCAAATATC	CA3	
Rerg	NM_001164214.1	1891-1990	TAATGGGTGAGTAAAATTCACTCTACGTGAACGAGCGTGCACAAAGTAGATGCTTGGCTGGCAAAAGGATTATGTAACTGCCAAGAGTTTATGATCCTTC	CA3	
Rgs14	NM_016758.3	2077-2176	GGCCTAGTTCCTTGGCATAAGCATAGTGAACACAAACGGGTGACTTTGGCAGTGCCAGCCTCCTCAACTTGTTCTAGGCTTTAGCAGGGGGCAGGAAGGA	CA2	
Rpl13a	NM_009438.5	751-850	GGGGACATTAGAGAGCTGACAGCGCACCACCTGCATGAGTTTTTATTAAAGTGCAAACCATGGGATGAATCAGTTGAGCTTCAGTGTTGAAAATGAGTAG	HK	
Rreb1	NM_001177868.1	895-994	CAGTCAGGCGATTTGGACAAGATGAGCGATGAAATCTTTCACTGCCCAGTGTGTTTCAAGGAGTTTGTTTGCAAGTATGAACTGGAGACCCACATGGAGA	DG	
Sdha	NM_023281.1	251-350	CTTGCGAGCTGCATTTGGCCTTTCTGAGGCAGGGTTTAATACTGCATGCCTTACAAAGCTCTTTCCTACCCGATCACATACTGTTGCAGCACAGGGAGGT	HK	
Slc39a6	NM_139143.3	671-770	GTGTTAATGTTGACTTGGCAGTTACCATGCAGCGACACCATCTGCAGCAGCTATTCTACCGCTACGGAGAGAATGATTCCTTGTCTGTTGAAGGCTTCAG	DG	
Slc6a7	NM_201353.1	3031-3130	CACCATGGGGATAGGAATGAGAAATGAGAGTTTTCTCCCTTGCCGCTGTGGCTTCCAATAACAATGGCTACCGAGACTCTGAGGTGGTCCACACGTCGGA	CA3	
Slc6a9	NM_008135.4	629-728	GTGGGCTATGGTATGATGGTGGTGTCCACATACATTGGCATCTACTACAACGTGGTCATCTGCATCGCCTTCTACTACTTCTTCTCGTCCATGACGCACG	OTHER	
Smoc2	NM_022315.2	1811-1910	CAACTTCACTTCCTCGTCCCTCTGTGGAGGGCCTGGTTTATTCCCTTGCCCTTTCCTCTGCCTGCAGTCTCCGTTGGCTTGCTCTCCGTGCTCTTGATGC	CA2/CA3	
Sorcs3	NM_025696.3	1869-1968	GTTGCCACGGGTAATATTGGCTCCGAACTCTCCTATACGGATATCGGTGTGTTCATCTCCTCTGATGGGGGCAACACATGGAGACAGATCTTTGATGAAG	CA1	
Spats2l	NM_001164566.1	191-290	CAGTTGTTCCGAACAAAAGCAATAATGAAATCGTGCTGGTACTCCAACAGTTTGATTTTAATGTGGATAAAGCCGTACAGGCCTTTGTGGATGGCAGTGC	CA1	
Spink8	NM_183136.2	403-502	AAAGTGCATGATGGGCCATGTGAACACTCCTCGGATGAGTCTGAACACTGATAAAAGTTACTGAGTTCTCAGAGAGGCACGTGGAGCACGACTGACTCTT	CA2/CA1	
Strip2	NM_177204.3	1759-1858	GCATGAAGCTAGGCATTGACGTGAACAGACACAAGGAAATCATTGTAAAGAGTATCTCTGCCCTGCTCCTTCTACTTCTCAAACACTTCAAACTCAACCA	CA2	
Syndig1l	NM_001033334.2	1105-1204	AAAGCTCAGAACTGTGTATTCTATGGAGCACCTTGGCCCTGAAGAGAACCTGCGACAATAGCTTGCTGAGGAGAGACCTCCCGTTCTGCAGCCCACACAG	CA2	
Tbp	NM_013684.3	71-170	GTGGCGGGTATCTGCTGGCGGTTTGGCTAGGTTTCTGCGGTCGCGTCATTTTCTCCGCAGTGCCCAGCATCACTATTTCATGGTGTGTGAAGATAACCCA	HK	
Tenm3	NM_011857.3	2586-2685	TCTATGACCGAATCAGTTTCCTGATTGGATCGGATAGCACCCACGTGCTCCCTGGAGAAAGTCCGTTCAATAAGAGTCTTGCGTCCGTCATCAGAGGCCA	CA1	
Thsd7a	NM_001164805.1	1827-1926	CTGGGGACCATGTACTTATGAAAACTGTAATGACCAGCAAGGCAAAAAAGGATTCAAATTAAGGAAGCGACGCATTACTAATGAGCCTACTGGAGGTTCT	DG	

Supplemental Table 6: Custom codeset for NanoString analysis (page 3 of 3)

*Genes that did not quantify above noise are designated in the Notes column. These genes were not included in analysis. HK = housekeeping gene

Gene	Accession #	Position	Target Sequence	Target	Notes
Tlr4	NM_021297.2	2511-2610	AACGGCAACTTGGACCTGAGGAGAACAAAACTCTGGGGCCTAAACCCAGTCTGTTTGCAATTAATAAATGCTACAGCTCACCTGGGGCTCTGCTATGGAC	OTHER	*
Trpc6	NM_013838.2	557-656	TCATTCAACAAGCCTGTCTATTGAGGAAGAACGCTTTCTAGATGCAGCTGAATATGGCAACATCCCAGTGGTGCGGAAGATGCTAGAAGAGTGTCATTCC	DG	
Trps1	NM_032000.2	9146-9245	CTTGGTTGGAGGGCACCATAAGAGTGTCAAGTATTATTATGTGGCCAAGGGGGTTATTTAAAACTCTGGTTCCCACGGGCCGAGAAAGTTTGGCATCATT	CA3	
Wfs1	NM_011716.2	2976-3075	CGCAGATCCTGCTGGAGGGTGGTTCTCTTTAGCACTGTCCACTTTGAATGCCGAGTGTCATAAGAAATTGCATGCTATCTTCACTCACAATCCTGCCCTT	CA1	
Ywhaz	NM_011740.2	456-555	AACGTTGTAGGAGCCCGTAGGTCATCGTGGAGGGTCGTCTCAAGTATTGAGCAGAAGACGGAAGGTGCTGAGAAAAAGCAGCAGATGGCTCGAGAATACA	HK	
Zfp804a	NM_175513.3	2823-2922	ACTCATCGCAGACTTCCAATGATCTTGCTACACCTGTGAATGTCACAAGGGACCCATCAAATAGCACCACTGACAATACCTTGCTTGAACACAATCAAAG	CA2	*
